# Biology and medicine in the landscape of quantum advantages

**DOI:** 10.1098/rsif.2022.0541

**Published:** 2022-11-30

**Authors:** Benjamin A. Cordier, Nicolas P. D. Sawaya, Gian Giacomo Guerreschi, Shannon K. McWeeney

**Affiliations:** ^1^ Department of Medical Informatics and Clinical Epidemiology, Oregon Health and Science University, Portland, OR 97202, USA; ^2^ Knight Cancer Institute, Oregon Health and Science University, Portland, OR 97202, USA; ^3^ Oregon Clinical and Translational Research Institute, Oregon Health and Science University, Portland, OR 97202, USA; ^4^ Intel Labs, Santa Clara, CA 95054, USA

**Keywords:** quantum computing, quantum advantage, computational biology, bioinformatics, medicine

## Abstract

Quantum computing holds substantial potential for applications in biology and medicine, spanning from the simulation of biomolecules to machine learning methods for subtyping cancers on the basis of clinical features. This potential is encapsulated by the concept of a quantum advantage, which is contingent on a reduction in the consumption of a computational resource, such as time, space or data. Here, we distill the concept of a quantum advantage into a simple framework to aid researchers in biology and medicine pursuing the development of quantum applications. We then apply this framework to a wide variety of computational problems relevant to these domains in an effort to (i) assess the potential of practical advantages in specific application areas and (ii) identify gaps that may be addressed with novel quantum approaches. In doing so, we provide an extensive survey of the intersection of biology and medicine with the current landscape of quantum algorithms and their potential advantages. While we endeavour to identify specific computational problems that may admit practical advantages throughout this work, the rapid pace of change in the fields of quantum computing, classical algorithms and biological research implies that this intersection will remain highly dynamic for the foreseeable future.

## 1. Introduction

### 1.1. Quantum computing: past to present

The notion that a *quantum computer* may be more powerful than a classical computer was first conceived some 40 years ago in the context of simulating physical systems [1–3]. Theoretical models of quantum computers quickly followed [4–6]. Then, in 1993, 11 years after Feynman’s talk on simulating physics [3], early formal evidence that a universal quantum computer may be more powerful than its classical counterpart arrived with proof of a *superpolynomial* quantum advantage on an artificial problem, recursive Fourier sampling [7]. This was shortly followed by the development of the first *quantum algorithm* for a practical problem, prime factorization, by Peter Shor in 1994—also yielding a superpolynomial advantage [8]. Shor’s algorithm was the first example of a quantum algorithm that could have significant real world implications by threatening the RSA cryptosystem [9], which is widely used to secure the Web. Its discovery initiated a flurry of research into quantum algorithms, a now burgeoning subfield of quantum information science (QIS), that has continued to the present.

More recently, in 2019, experimental evidence of the increased computational power of quantum computers was provided via the first successful quantum supremacy^1^ experiment on a 53 qubit superconducting device [10]. Similar experiments leveraging various quantum hardware technologies have since followed [11–13]. While it has been debated whether these experiments represent true examples of quantum supremacy,^2^ they have nonetheless galvanized the QIS field around the practical potential of quantum computers in the near term.

Quantum hardware has now entered the *noisy intermediate-scale quantum* (NISQ) era [14], a stage of maturity characterized by devices with low qubit counts, high error rates, and correspondingly short coherence times. While it is unclear how long the transition from the NISQ era towards *fault tolerant quantum computation* (FTQC) will take—whether it will occur by error correction [15] or inherently fault tolerant hardware based on topological properties [16]—estimates range from several years to several decades. Yet, with NISQ devices moving from the laboratory to the cloud, now is an opportune time for computationalists in biology and medicine to begin exploring the value that quantum approaches may bring to their research toolbox.^3^

### 1.2. Biology and medicine as computational sciences

Over the past three decades, biology and medicine have evolved into highly quantitative fields [17]. Areas of inquiry span from foundational questions on the origins of life [18] and the relationships between protein structure and biological function [19] to ones with a direct impact on clinical practice, such as those concerned with the oncogenesis of cancer [20,21], the development of novel drugs [22] and the precise targeting of therapeutics on the basis of genetic mutations [23] and other clinical indicators [24]. However, despite the substantial progress facilitated by computational methods and the expansion of *high performance computing* (HPC) environments, fundamental constraints persist when modelling biological and clinical systems.

System complexity is one example. This constraint arises from both the first-order biological complexity, as can be seen in the metabolic processes of individual cells [25] or the binding of protein receptors to ligands [26], and higher-order clinical complexity, occurring at the intersection of complex biological, behavioural, socioeconomic, cultural and environmental factors [27]. On the one hand, this system complexity has made biological and clinical research a verdant playground for the development of many novel, efficient computational algorithms and approaches. On the other hand, practical algorithms typically manage system complexity via reductionist frameworks. A consequence of this is that existing computational models often fail to capture and reconcile important system dynamics. Perhaps the clearest examples exist in the fields of structural biology and biochemistry, where even simple biological molecules, such as proteins, ligands and transcription factors regularly challenge classical heuristic methods developed to model their structure and binding behaviours. Quantum computers, if sufficiently robust ones can be built, promise to fundamentally reduce the *algorithmic complexity* of constructing and analysing many of these models. This may allow solutions to hard computational problems to be computed with greater algorithmic efficiency, which could in turn reduce compute times and improve the fidelity of practical models.

The second constraint is one of scale. Looking to healthcare alone, as much as 153 exabytes of data were generated in 2013 with a projected annual growth rate of 48% [28]. Extrapolating this growth rate, it is plausible that over 2300 exabytes were generated in 2020. Similar data challenges also exist in biology. For example, the massive growth of high-throughput sequencing has led to exabytes of highly complex genomic, epigenomic, transcriptomic, proteomic and metabolomic data types (among others). To manage these large data volumes, centralized data repositories have proliferated (e.g. see the ever-growing dbGaP [29] or the more recent Genome Data Commons [30]). These massive data resources are crucial to the re-use of high-value data in secondary analyses and reproducibility studies. However, even with the wide use of HPC infrastructures, large bioinformatics and computational biology workflows often extend for days, weeks, or longer. In recent years, this challenge has grown with the expansion of other areas demanding significant computational *resources*. Examples include high-resolution imaging (e.g. *cryo-EM*) and massive deep learning inference pipelines with of the order of 10^9^ (or greater) model parameters. While it is not anticipated that scalability constraints will be addressed by quantum computing technologies in the near term, FTQC devices may offer a partial solution to some of these challenges over the long term.

### 1.3. Approach

Given these challenges across biology and medicine and the potential of quantum computing, a common question among domain computationalists interested in applying quantum computing is: ‘When will I be able to leverage quantum computing for [insert preferred application]?’ The answer to this question is complex and can be factored into a number of considerations:

— How can it be ascertained whether a problem will benefit from a quantum advantage?
— What scale of problem instance is required to meaningfully demonstrate such an advantage?
— What hardware and software are required to translate a quantum advantage from theory to practice?
— How can a practical quantum advantage be detected and measured once it has been achieved?

While prior work [31–35] has considered aspects of these questions for a variety of application areas (e.g. genomics, drug-design, clinical phenotyping, neurimaging), a basic framework for comprehending the potential of quantum computing on specific problems and application areas has yet to be clearly articulated. In this work, we endeavour to fill this gap by defining such a framework, the landscape of quantum advantages, which integrates complexity theoretic results, context-based evidence levels for quantum advantages, and known hardware constraints (§3). Next, we expand our analysis to consider in detail a wide variety of quantum approaches developed for near-term quantum computers without error correction or fault tolerance (§4). We then consider an array of specific applications across biology and medicine and their prospects for yielding practical quantum advantages (§5). Finally, we conclude with a summary of this work and the future prospects of quantum computing in biology and medicine (§6).

### 1.4. Impact

It is our hope that this work can serve as both (i) a resource for computationalists in biology and medicine interested in developing quantum computing proof of principles for their field in the NISQ era and (ii) a guide for quantum algorithms researchers interested in developing algorithms targeting applications in biology and medicine. As such, we have written this perspective with an interdisciplinary audience in mind and endeavoured to minimize the need for a formal background in biology, medicine, quantum information science and computational complexity. However, given the often counterintuitive nature of the physical principles behind quantum computing, we begin in §2 with a brief primer on quantum computing. Further, we have included in the electronic supplementary material the following resources: (i) a glossary of key terms, (ii) an overview of Dirac notation (a commonly used mathematical notation for quantum information) [36], and (iii) the established axioms of quantum information. If additional background is desired by readers, we refer them to the resources highlighted in this endnote.^4^

## 2. A brief primer on quantum computing

Here we provide a brief overview of quantum computing using Dirac notation [36], which we describe in the electronic supplementary material. For a thorough treatment of quantum computing fundamentals, see the standard textbook for the QIS field by Nielsen & Chuang [37].

### 2.1. Qubits

A qubit is a two-dimensional quantum system with a defined computational basis. Multiple formalisms exist for representing a qubit. In the geometric representation, a qubit is fully specified as

$$|\psi \rangle =e^{i\gamma }\left(\cos\frac{\theta }{2}|0\rangle +e^{i\rho }\sin\frac{\theta }{2}|1\rangle \right),$$

where *γ*, *ρ* and *θ* are real-valued parameters. The terms e^i*γ*^ and e^i*ρ*^ are known as the global and relative phase of the qubit, respectively. Typically, the global phase is not of interest and is discarded. This leads to the following classic description of a qubit in the geometric representation:

$$|\psi \rangle =\cos\frac{\theta }{2}|0\rangle +\;e^{i\rho }\sin\frac{\theta }{2}|1\rangle ,$$

where *θ* and *ρ* define a point on the unit sphere. Notably,

$$|0\rangle =\left[\begin{array}{c} 0\\ 1 \end{array}\right]\;\mathrm{and}\;|1\rangle =\left[\begin{array}{c} 1\\ 0 \end{array}\right]$$

indicate the computational basis (in this case, the standard basis {|0〉, |1〉}). The computational basis often implies the appropriate measurement basis for the qubit.

In quantum information theory, a qubit is often simplified to the following information-theoretic representation:

|ψ⟩=α|0⟩+β|1⟩.

Here, the values *α* and *β* represent probability amplitudes—a generalization of probabilities from real to complex values. These can be viewed as the core mechanism by which a qubit stores information. This leads to perhaps the simplest representation of a qubit,

$$|\psi \rangle =\left[\begin{array}{c} \alpha \\ \beta \end{array}\right],$$

as a two-dimensional vector of complex values.

### 2.2. Measuring a qubit

Measuring a qubit yields a classical post-measurement state and destroys its quantum state in the process. To measure a qubit, we must first define a *Hermitian matrix* known as a projector. For a single qubit in the standard basis, we can define the following two projectors via the outer product:

$$P_{0}=|0\rangle \langle 0|=\left[\begin{array}{cc} 1 & 0\\ 0 & 0 \end{array}\right]\;\mathrm{and}\;P_{1}=|1\rangle \langle 1|=\left[\begin{array}{cc} 0 & 0\\ 0 & 1 \end{array}\right].$$

The Born rule [38] states that the probability of measuring each basis state corresponds to the magnitude-squared of their probability amplitude,

$$|\alpha {|}^{2}+|\beta {|}^{2}=1,$$

thus, given the above projectors, the probability of measuring |*i*〉 is *p*(|*i*〉) = 〈*ψ*|*P*_*i*_|*ψ*〉. Finally, it is important to note that following its measurement, the qubit will remain in the classical state |*i*〉.

### 2.3. Properties of quantum information

Quantum computers benefit from the combination of three properties—*superposition*, *entanglement* and *quantum interference*. From the physical perspective, the combination of these three properties is unique to quantum mechanical systems. Both superposition and entanglement only become apparent when examining multi-qubit states.

#### 2.3.1. Superposition

Multiple qubits can be expressed as a linear combination over a basis set, known as a coherent superposition. For example, in the case of two qubits in the standard basis,

|ψ⟩=α|00⟩+β|01⟩+γ|10⟩+δ|11⟩.

In general, *n*-qubit states are represented as a state vector occupying a *Hilbert space* with 2^*n*^ dimensions.

#### 2.3.2. Entanglement

Multiple qubits can also be entangled. In the context of computation, entanglement is a computational resource that is unique to quantum systems and has been identified as the source of multiple theoretical quantum advantages.

Perhaps the best-known examples of entanglement are the set of four 2-qubit Bell states, $\left\{|\Phi^{+}\rangle ,|\Phi^{-}\rangle ,|\Psi^{+}\rangle ,|\Psi^{-}\rangle \right\}$, which represent the four maximally entangled states for two qubits [39]. For example, given the Bell state

$$|\Phi^{+}\rangle =\frac{1}{\sqrt{2}}(|00\rangle +|11\rangle ),$$

a measurement of the state of the first qubit immediately implies knowledge of the state of the second qubit. This can be seen by expanding the Bell state,

$$|\Phi^{+}\rangle =\frac{1}{\sqrt{2}}|00\rangle +\frac{0}{\sqrt{2}}|01\rangle +\frac{0}{\sqrt{2}}|10\rangle +\frac{1}{\sqrt{2}}|11\rangle ,$$

and noting the zero values in the numerator of the probability amplitudes for the |01〉 and |10〉 basis states.

Following the Born rule, we see that the probabilities of observing the |00〉 and |11〉 states are $p(|00\rangle )=|1/\sqrt{2}{|}^{2}=1/2$ and $p(|11\rangle )=|1/\sqrt{2}{|}^{2}=1/2$. It thus follows that while a measurement of one qubit has a 1/2 probability to yield either a |0〉 or |1〉, the measurement of the second qubit is guaranteed to yield a basis state identical to that of the first qubit. Looking to the pre-measurement quantum state, $\left|\Phi^{+}\right\rangle$, this fact is made clear given that both $p(|01\rangle )=|0/\sqrt{2}{|}^{2}=0$ and $p(|10\rangle )=|0/\sqrt{2}{|}^{2}=0$.

#### 2.3.3. Interference

Quantum interference results when probability amplitudes between two quantum states are summed or subtracted, which results in constructive and destructive interference, respectively.

Perhaps the most clear example exists in the repeated application of a single-qubit quantum gate, known as the Hadamard gate,

$$H=\frac{1}{\sqrt{2}}\left[\begin{array}{cc} 1 & 1\\ 1 & -1 \end{array}\right].$$

In particular, given an application of the Hadamard gate on the input state |0〉, we will then have the state

$$H|0\rangle =\frac{1}{\sqrt{2}}(|0\rangle +|1\rangle ).$$

This state is an equal superposition with a relative phase of 0. If we then apply the Hadamard gate again, we will end up with the state

$$H\frac{1}{\sqrt{2}}(|0\rangle +|1\rangle )=\frac{1}{2}(|0\rangle +|1\rangle )+\frac{1}{2}(|0\rangle -|1\rangle )=|0\rangle .$$

Here, we see that the + and − arithmetic operations on the probability amplitudes result in them cancelling each other out, providing an example of destructive interference.

### 2.4. Quantum gates

Quantum logic gates are unitary *operators* that can be represented as square matrices, and as shown in the interference example above, implement a form of *reversible computation*.

#### 2.4.1. Single qubit gates

Most well known among the single qubit gates are the identity gate and single qubit Pauli matrices:

$$I=\left[\begin{array}{cc} 1 & 0\\ 0 & 1 \end{array}\right]\;\mathrm{and}\;\sigma_{x}=\left[\begin{array}{cc} 0 & 1\\ 1 & 0 \end{array}\right]$$

and

$$\sigma_{y}=\left[\begin{array}{cc} 0 & -i\\ i & 0 \end{array}\right]\;\mathrm{and}\;\sigma_{z}=\left[\begin{array}{cc} 1 & 0\\ 0 & -1 \end{array}\right].$$

Any single qubit gate can be written as a linear combination of Pauli matrices.

#### 2.4.2. Multi-qubit gates

Multi-qubit gates act on two or more qubits. Among these are the controlled gates, such as the two qubit controlled-NOT or CNOT,

$$\text{CNOT}=\left[\begin{array}{cccc} 1 & 0 & 0 & 0\\ 0 & 1 & 0 & 0\\ 0 & 0 & 0 & 1\\ 0 & 0 & 1 & 0 \end{array}\right].$$

Controlled gates implement a form of conditional logic. For example, the CNOT gate applies a NOT gate to the second qubit if and only if the first qubit is in the |1〉 state. For example, given the state |10〉,

CNOT|10⟩=|11⟩.

Non-conditional gates also exist. Of special practical interest is the *SWAP gate*, which can be used to swap the quantum state between two qubits:

$$\text{SWAP}=\left[\begin{array}{cccc} 1 & 0 & 0 & 0\\ 0 & 0 & 1 & 0\\ 0 & 1 & 0 & 0\\ 0 & 0 & 0 & 1 \end{array}\right].$$

For example, given the state |10〉,

SWAP|10⟩=|01⟩.

This special property makes the SWAP gate and its variants, such as the *i*SWAP, particularly useful for routing quantum information during a computation.

While the above quantum logic gates are discrete, in general they need not be. If we recall the geometric representation, a quantum gate may apply any arbitrary rotation(s) to the target qubit(s).

## 3. The landscape of quantum advantages

*The landscape of quantum advantages* is a set of concepts that together are central to the identification, characterization and realization of quantum advantages. These concepts, which we present below, include a classification scheme for quantum advantages, context-based evidence levels for establishing their existence and practical realization, and known hardware constraints that can influence their practical implementation.

### 3.1. Theoretical quantum advantages

How can we define a quantum advantage from the theoretical perspective? While multiple mathematical formulations have been described in the literature (e.g. [40,41]), for our purposes, we simply state that a theoretical quantum advantage is defined by four key properties:

(1) *Problem*: a formal computational problem.
(2) *Algorithms*: a classical algorithm and a quantum algorithm, each of which solve the computational problem.
(3) *Resources*: one or more resources, such as time, space, or data, that are consumed by both the classical and quantum algorithms.
(4) *Bounds*: analytical bounds on the resource consumption (e.g. a worst-case time complexity bound) for both the classical and quantum algorithms.^5^

A range of theoretical quantum advantages have been identified. For example, for the general problem of unstructured search, *Grover’s algorithm* [42,43] yields a quadratic advantage with a query complexity of $O(\sqrt{N})$ relative to the *O*(*N*) queries required classically^6^ (in this context, a query can be thought of as a function call). On the other end of the spectrum, the *k-Forrelation* problem [44,45] admits a quantum algorithm with the largest known (superpolynomial) quantum advantage—where $\tilde{\Omega }(N^{1-1/k})$ queries are required by a classical randomized algorithm (omitting logarithmic factors^7^), only ⌈k/2⌉ queries are required by the quantum algorithm, where ⌈⋅⌉ indicates a ceiling function rounding up to the greatest integer. In most cases, theoretical quantum advantages should be thought of as loose approximations of the degree of quantum advantage that may be possible in practice.

### 3.2. Classifying theoretical advantages

**Figure 1.**
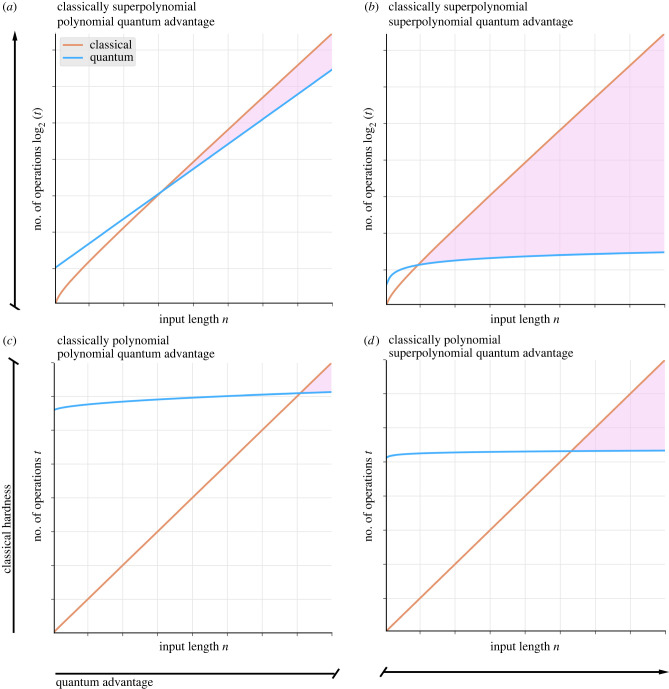
Classifying quantum advantages. A conceptual illustration of four classes of quantum advantage, which we define across two axes: (i) the classical resource consumption (vertical, where *t* represents a time-based resource, such as the number of gate operations required) and (ii) the strength of the advantage (horizontal). On fault tolerant hardware, quantum advantages are expected to have variable computational overheads (see §3.4 for details); as such, the pink region indicates where the quantum advantage begins (i.e. where the quantum computational overhead is amortized). (*a*) Polynomial advantages on classically hard problems. Amplitude amplification and estimation [46] provides a general framework to achieve polynomial quantum advantages on search and optimization problems. These techniques have led to quantum versions of dynamic programming algorithms [47,48], which are relevant to many tasks in genomics, such as sequence alignment. (*b*) Superpolynomial advantages on classically hard problems. *Hamiltonian* simulations of strongly correlated fermionic systems are known to be classically hard. Many quantum Hamiltonian simulation algorithms yield superpolynomial advantages [49] and may lead to substantial improvements in drug discovery pipelines [32]. (*d*) Superpolynomial advantages on classically easy problems. Matrix inversion is among a set of linear algebra subroutines central to machine learning. In particular, inverting a feature covariance matrix is common to many classical machine learning approaches, such as support vector machines and Gaussian processes. The quantum linear systems algorithm (QLSA) [50] performs this crucial subroutine for a variety of quantum machine learning algorithms (e.g. [51,52]) and runs in polylogarithmic time relative to the matrix dimensions. While this implies a superpolynomial advantage (omitting the complexity due to the condition number), practical realizations are expected to depend on matrix rank and may require the development of quantum random access memory (QRAM) [53–57]. (*c*) Polynomial advantages on classically easy problems. Grover’s algorithm [42] for unstructured search is the classic example in this class. It, and similar quantum algorithms, may be used as subroutines in conjunction with other quantum algorithms that offer greater advantages.

A theoretical quantum advantage can be classified on the basis of two factors (figure 1). The first one relates to the classical hardness of the computational problem, which is defined by the best-known classical algorithm^8^ or a provable upper bound. In particular, a computational problem may be classified as *easy* or *hard* according to whether its classical algorithmic complexity (typically for a worst case input) is *polynomial* or superpolynomial, respectively [58]. The second factor relates to the size of the advantage yielded by the quantum algorithm relative to the classical algorithm. Often, these advantages result from a reduction in complexity for a computational resource. Like algorithmic complexity, the size of an advantage may also be classified as polynomial or superpolynomial. Here, we would like to make clear that the terms ‘easy’ and ‘hard’ refer only to classical computability; they are not intended to refer to the attainability of a practical quantum advantage or the difficulty of implementing a quantum approach.

For the rest of this subsection, we discuss paradigmatic examples of each class of quantum advantage. While we briefly highlight some relevant algorithms and applications in biology and medicine, we provide a much more detailed account of potential applications in §5.

#### 3.2.1. Polynomial advantages for easy problems

The most well-known algorithm in this class is Grover’s algorithm for unstructured search, which has a worst-case complexity of $O(\sqrt{N})$ under the *query model* relative to the *O*(*N*) complexity of its classical counterpart [42]. At its core, Grover’s algorithm uses *amplitude amplification* [46], a general quantum algorithm technique that yields polynomial advantages without requiring a specific problem structure. Many other quantum algorithms also fall under this class of quantum advantage. These include ones for convex optimization [59,60], semi-definite programming [61] and calculating graph properties (e.g. bipartiteness, st-connectivity and cliques) [62–64]. From the perspective of biology and medicine, some problems in network and systems biology can be cast as convex optimization or network inference problems incorporating graph property testing.^9^ Practical examples include inferring the characteristics and behaviours of gene regulatory, protein interaction and metabolic networks.

#### 3.2.2. Polynomial advantages for hard problems

These include many *optimization* variants of *NP-hard*^10^ problems that may benefit from amplitude amplification. Examples of quantum algorithms in this class include ones for constraint satisfaction [65] and combinatorial optimization [47,48,66]. Notably, algorithms in this class were among the first to target applications specific to biology and medicine, including sequence alignment [67–69] and the inference of phylogenetic trees [70]. Sequence alignment, in particular, represents a crucial computational primitive for many tasks in bioinformatics and computational biology.

#### 3.2.3. Superpolynomial advantages for easy problems

While no algorithms specific to problems in biology or medicine are known to exhibit this class of advantage, a number of quantum machine learning (QML) algorithms with general relevance do. Perhaps the most prominent example is the quantum linear systems algorithm (QLSA) [50] for high-rank matrices, which led to the development of many early QML algorithms. Another example is a quantum algorithm for pattern matching [71], which yields a superpolynomial advantage as the size of the pattern approaches the size of the input. Quantum algorithms in this class typically exploit compact quantum data encodings, such as amplitude encoding, that allow for certain computations to be performed using a polylogarithmic number of qubits and operations relative to their comparable classical algorithms. However, algorithms in this class may also be subject to several practical constraints related to data input and output sampling [72], which we discuss in detail in §3.4.

#### 3.2.4. Superpolynomial advantages for hard problems

The most widely known example of an algorithm in this class may be Shor’s algorithm for factoring composite integers into their primes [73], a problem that has no direct relevance to biology or medicine. Quantum algorithms for simulating quantum physics [74–82], however, may find significant applications. In principle, these algorithms could provide superpolynomial advantages for a vast range of hard problems related to simulating physical systems.^11^ Examples include characterizing the ground states of biologically relevant small molecules, the behaviour of chemical solutions, and the ternary and quaternary structures of biological molecules [83]. In general, this is the most desirable class of quantum advantage; however, known examples are dependent on specific problem structures that are uncommon among practical computational problems [84].

### 3.3. Context-based evidence for quantum advantages

A quantum advantage may be supported by multiple forms of evidence that can vary by its context. At the highest level, these contexts can be theoretical or empirical. In the theoretical context, abstract evidence is collected to answer whether a quantum algorithm can yield an advantage over the most efficient classical algorithm. By contrast, empirical evidence is collected to answer whether a specific quantum algorithm and device can, together, yield an observable advantage over a classical approach given a well-defined problem and context-dependent metric. Importantly, while an experimental advantage may also be practical, evidence from an operational setting is best suited to addressing questions around the practical value of an empirical quantum advantage. In this section, we describe these contexts in detail and their implications for the evidence required to establish a quantum advantage.

#### 3.3.1. Theoretical advantages

These result from an improvement in analytical bounds on resource efficiency by a quantum algorithm relative to a well-motivated classical counterpart. The core resource in question typically involves units of time, space or information (e.g. samples from an experiment). In practice, these units of comparison may be gates, queries, bits or error rates. Theoretical advantages are defined mathematically, may be conjectured, and are often contingent on well-founded assumptions from computational complexity theory. Crucially, they need not be application-oriented.^12^ Instead, they may be conceptual—designed with the express intent of demonstrating that an advantage exists for an artificial task or broader class of problems that have theoretical interest. Examples of conceptual quantum algorithms yielding theoretical advantages include the Deutsch–Jozsa algorithm [85], Bernstein–Vazirani algorithm [86], Simon’s algorithm [87] and one for solving the Forrelation problem [88]. Despite their theoretical motivation, practical applications may nonetheless follow from conceptual algorithms. One example of this can be seen with Shor’s algorithm for prime factorization [8], which was inspired by Simon’s algorithm [87].

#### 3.3.2. Experimental quantum advantages

These are a type of empirical quantum advantage observed in an experimental context. Crucially, they require the comparative analysis of computational metrics that precisely measure the advantage in question.^13^ To facilitate these comparisons, these metrics are ideally defined over both quantum and classical approaches. While theoretical evidence (i.e. proofs, conjectures and numerical models) may support an experimental advantage *a priori*, it is the computational benchmarking of an implementation that serves as the evidence for establishing an experimental quantum advantage. For instance, the first quantum supremacy experiment [10] represented a real-world demonstration of an experimental quantum advantage. First, a theoretical advantage was proven and numerically estimated [89], then initial experimental work soon followed, which yielded the first hint of such an experimental advantage [90]. Finally, this culminated in an experimental demonstration of the quantum advantage—as evidenced by substantial cross-entropy benchmarking [10]. Similar trajectories are observable for other quantum supremacy experiments, such as one recently demonstrated on a non-universal photonics-based device [11,91,92].

#### 3.3.3. Operational quantum advantages

These result from the successful translation of an experimental advantage to an applied setting. As such, in addition to the computational benchmarking required to validate the experimental advantage, this type of advantage may require the definition and measurement of an operational metric—a key performance indicator (KPI)—to gauge the extent of the practical advantage when deployed. Accordingly, this type of advantage considers the greater context outside of the computational environment. In particular, it can be viewed as one that additionally integrates the organizational, economic and social context of the target application.^14^ Challenges to realizing an operational quantum advantage may include organizational inertia, entrenched support for incumbent classical methods, and difficulties in the integration of the experimental quantum advantage into existing software, hardware and network infrastructure. To date, no obvious demonstration of an operational quantum advantage has occurred and estimates of when such an advantage may occur vary greatly.

#### 3.3.4. From theory to practice

The context of a quantum advantage is used to determine what evidence is required to establish it. While theoretical evidence has historically preceded experimental evidence, the chronological order of evidence levels may vary. This is expected to become increasingly apparent as interest in practical quantum approaches and access to near-term quantum hardware expands. In the absence of supporting theoretical evidence, the pursuit of experimental advantages may be motivated instead by expert intuition (e.g. around the structure of a computational problem and how quantum information may be beneficial to finding its solution). While such an intuition-based approach provides practical flexibility, it should be cautioned that it may, too, be a source of bias [93]. To manage this risk for specific applications, robust benchmarking and the publication of full experimental evidence (e.g. data and analysis code) through open science tools will be key to community verification of claimed empirical quantum advantages over the near term, especially in operational contexts.

### 3.4. Practical constraints on theoretical advantages

Translating a theoretical advantage into an experimental one is often challenging. These challenges largely arise from the practical constraints of NISQ hardware. In this section, we discuss some of these constraints and how they inform the translation of theory into practice. For an overview of the potential feasibility of a variety of quantum advantages with respect to quantum hardware, see table 1.

**Table 1.** Selected theoretical quantum advantages and their expected feasibility by hardware maturity. The type of theoretical advantage yielded by a quantum algorithm informs the degree of hardware maturity that is expected to be required for its practical realization. Whereas superpolynomial quantum advantages on classically hard problems may be attained by NISQ devices in the coming years, other forms of advantage (i.e. superpolynomial advantages on classically easy problems and polynomial advantages on both classically easy and hard problems) will likely require far greater hardware maturity (i.e. FTQC). In the right-most columns, we indicate the likelihood of the stated quantum advantage to be realized by a practical application on nisq and ftqc hardware (i.e. very unlikely, unlikely, possibly, likely, very likely). These likelihoods were assessed via (i) a synthesis of the current state of the literature at the time of writing and (ii) discussions with researchers in the QIS field. Importantly, they are intended only to provide coarse-grained guidance. Note that sample complexity advantages on quantum data are supported by theoretical proofs and are conjectured for classical data; algorithms with the potential to demonstrate them in practice are currently being investigated.

quantum advantage	resource	quantum complexity	classical complexity	example application	classical constraint	nisq	ftqc
super-polynomial	operations	polynomial	super-polynomial	Hamiltonian simulation	complexity	likely	very likely
super-polynomial	operations	poly-logarithmic	polynomial	matrix inversion	very large data	possibly	likely
polynomial	queries	polynomial	polynomial	unstructured search	very large data	very unlikely	possibly
polynomial	queries	super-polynomial	super-polynomial	dynamic programming	complexity	unlikely	possibly
polynomial	samples	polynomial	polynomial	machine learning	sample generation	possibly	possibly
super-polynomial	samples	polynomial	super-polynomial	machine learning	complexity; quantum data	unlikely	possibly

#### 3.4.1. Logical versus noisy qubits

Theoretical quantum algorithms tend to be modelled with FTQC devices—ones implementing logical qubits upon which error-free gates, state preparation and measurement are performed. Current hardware remains far from such a scalable device. Instead, existing NISQ devices have dozens to hundreds of error-prone qubits. The error characteristics of quantum devices arise from a number of error types. These include state preparation and measurement (SPAM) error, gate (operation) errors, emergent errors, such as cross-talk [94–96] and systematic errors due to device calibration or fabrication defects. While these errors can in principle be factored into their constituent phase and bit-flip components, the heterogeneity of their generating processes will likely require an all of the above approach to realize scalable FTQCs with coherence times sufficiently long to run large quantum circuits with polynomial depth.

Approaches to realizing FTQC are expected to require error mitigation techniques^15^ (e.g. [97–109]) to bring hardware error rates below a fault-tolerance threshold [15]. Below this threshold, it may be possible to use error correcting codes (e.g. see the CSS code [110,111], surface code [112], XZZX surface code [113] and honeycomb code [114]) to enable practically unlimited coherence times and resilience to environmental noise.^16^

Numerical simulations of error correction approaches imply that the overheads incurred by some of these strategies will place limits on the class of quantum advantages that can be achieved for many years.^17^ Indeed, some polynomial advantages [41] and even superpolynomial advantages [115] using the surface code may require very large numbers of qubits to encode the necessary logical qubits. Fortunately, the rapid pace of progress and many avenues being explored give much reason for optimism. One recent example lies in a numerical simulation of the honeycomb code [114], which indicated that achieving computations on circuits with trillions of logical operations may be possible with as few as 600 qubits [116], given modest assumptions around qubit error rates.

#### 3.4.2. Qubit connectivity

With few exceptions, NISQ devices are expected to have limited qubit connectivity. By contrast, the mathematical proofs behind theoretical quantum advantages often assume all-to-all connectivity within qubit registers. Fortunately, a growing body of evidence [117,118] around the number of SWAP gates [119,120] required to emulate all-to-all connectivity on lower connectivity architectures indicates this may only present a soft constraint on practical quantum algorithms. For example, one recent numerical simulation demonstrated that all-to-all connectivity can be simulated with a two-dimensional grid architecture (with logarithmic or sublinear bounds on the overhead) for three quantum subroutines central to quantum simulation, machine learning and optimization algorithms [121]. This work also highlighted that logarithmic bounds may also be realized in architectures with even greater connectivity constraints provided sufficient additional qubits are available.^18^ Altogether, these numerical simulations imply that qubit connectivity on its own may present only a mild constraint when pursing practical quantum advantages.

#### 3.4.3. Input constraints

Theoretical quantum algorithms typically use a combination of abstract input models. These input models can be either *oracles*—‘black-box’ functions for which the form of the input and output is known but the implementation of the function is not—or data inputs. Both oracles and data inputs can be either quantum or classical. Practically, oracles can be viewed as algorithm subroutines or queryable data structures. Importantly, the type of oracle (i.e. quantum or classical) can either improve or limit the feasibility of implementing a quantum algorithm on an NISQ device. Whereas quantum oracles may place additional complexity requirements with respect to the number of qubits or gates required to implement a quantum circuit, classical oracles may mitigate the size of a quantum circuit by offloading classically efficient computations to a classical device. Similarly, the input of data typically requires either the encoding of classical information into qubits (table 2) or the loading of a quantum state. These data input steps generally demand additional circuit depth.

**Table 2.** Quantum data encoding methods. Many methods exist for encoding data into quantum algorithms. For illustrative purposes, we use example data (*top*), which we notate as an *n* by *m* matrix, *X*. Using these example data, we highlight three commonly used quantum data encodings (*bottom*): (i) *Basis encoding* encodes *X* as a superposition of *n* computational basis states of *mk* qubits, where *k* is the number of qubits necessary to represent the feature values. Given the example data, *k* = 3 + 1 is sufficient for the range [ − 7, 7] (i.e. 3 qubits encode the value in the range [0, 7] and 1 qubit encodes the parity). In basis encoding, each unique value of *x*_*i*_ is associated with a discrete basis state, which results in a sparse encoding. (ii) *Angle encoding* allows for the encoding of *X* into *nm* qubits by parametrizing each qubit with a real value, *x*_*ij*_, and then computing the tensor product. This results in a continuous encoding where each value of *X* is encoded independently as an angle into each qubit. (iii) *Amplitude encoding* allows for the input of *X* into the amplitudes of a quantum system. This results in a very compact encoding allowing *nm* values (i.e. every feature) to be encoded into the amplitudes of log(*nm*) qubits. Unlike angle encoding, this results in a continuous embedding of the values of *X* into the quantum state (i.e. in general, the values of *X* cannot be made independent of one another). For hybrid quantum–classical algorithms (discussed in §4), the type of data encoding method used can significantly influence both the expressivity [122,123] of the parametrized quantum circuit [124] and the appropriate procedure for sampling its output distribution.

sample *i*	feature 1 $x_{i_{1}}$	feature 2 $x_{i_{2}}$	binary/basis encoded $\left(x_{i_{2}},x_{i_{1}}\right)$	angle encoded $\left(x_{i_{1}},x_{i_{2}}\right)$	amplitude encoded $\left(x_{i_{1}},x_{i_{2}}\right)$
1	3	2	0010 0011	3*π*/4, 2*π*/4	$3\sqrt{141},3\sqrt{141}$
2	1	−5	1101 0001	1*π*/4, − 5*π*/4	$1\sqrt{141},-5\sqrt{141}$
3	7	2	0010 0111	7*π*/4, 2*π*/4	$7\sqrt{141},2\sqrt{141}$
4	0	−7	1111 0000	0*π*/4, − 7*π*/4	$0\sqrt{141},-7\sqrt{141}$

encoding	expression	example			
basis encoding	$\left|X\rangle =\frac{1}{\sqrt{N}}\sum_{i=1}^{N}|x_{i}\right\rangle$	$\left|X\rangle =\frac{1}{2}|1111\;0000\rangle +\frac{1}{2}|1101\;0001\rangle +\frac{1}{2}|0010\;0111\rangle +\frac{1}{2}|0010\;0011\right\rangle$			
angle encoding	$\left|X\rangle =\overset{N}{\underset{i=1}{⨂}}\cos(x_{i})|0\rangle +\sin(x_{i})|1\right\rangle$	$|X\rangle =\left[\begin{array}{c} \cos(3\pi /4)|0\rangle \\ \sin(3\pi /4)|1\rangle \end{array}\right]⊗\left[\begin{array}{c} \cos(2\pi /4)|0\rangle \\ \sin(2\pi /4)|1\rangle \end{array}\right]⊗\cdots ⊗\left[\begin{array}{c} \cos(0\pi /4)|0\rangle \\ \sin(0\pi /4)|1\rangle \end{array}\right]⊗\left[\begin{array}{c} \cos(-7\pi /4)|0\rangle \\ \sin(-7\pi /4)|1\rangle \end{array}\right]$			
amplitude encoding	$\left|X\rangle =\sum_{i=1}^{N}\frac{x_{i}}{\sqrt{\|X{\|}^{2}}}|i\right\rangle$	$\left|X\rangle =\frac{3}{\sqrt{141}}|000\rangle +\frac{2}{\sqrt{141}}|001\rangle +\cdots +\frac{0}{\sqrt{141}}|110\rangle -\frac{7}{\sqrt{141}}|111\right\rangle$			

When theoretically analysing quantum algorithms, the complexity overheads of oracles and data input are sometimes omitted from the stated complexity. Despite these omissions, these subroutines can strongly influence whether an empirical quantum advantage is possible. One prominent example exists for the class of superpolynomial advantages on classically easy problems.^19^ In principle, for this class, the data input must be performed in *O*(polylog(*n*)) to maintain the quantum advantage, a point highlighted in a discussion [72] of the potential limitations of the QLSA.^20,^^21^

Many quantum algorithms for preparing initial quantum states already exist [125–129]. Nonetheless, over the near term, low qubit counts and circuit depths are expected to limit the realization of many quantum advantages in practice. To mitigate this challenge, a variety of approaches have been proposed. These include the ‘coresets’ input model [130], a technique with conceptual similarities to importance sampling, for which one recent practical implementation has been demonstrated [131]. Similarly, data transformations designed to compress the correlation structure of features into a lower dimensional space may also prove useful. Further, *hybrid quantum–classical approaches* (discussed in detail in §4) and recent work highlighting the relationship between data encodings and learned feature embeddings [123,132,133] have substantially improved our understanding of efficient data input for near-term devices. In particular, hybrid quantum–classical techniques can allow for substantial reductions in circuit width and depth for many applications [134]. Thus, while input constraints are expected to remain prevalent in the near term (particularly for algorithms requiring polynomial qubits and circuit depths), the substantial work on mitigating them and recent progress with hybrid approaches provides a growing toolkit for managing them.

#### 3.4.4. Output constraints

These can largely be attributed to two highly related factors arising from the physics of quantum information. The first is Holevo’s bound [135], a theoretical result that limits the amount of information that can be efficiently retrieved from *n* qubits to *n* classical bits. Naively, this implies that the size of a solution output by a quantum circuit must be (at most) polynomial in the number of qubits. The second constraint relates to the probabilistic nature of quantum information. In particular, even if a solution can be output within the space afforded by Holevo’s bound, the number of quantum circuit evaluations (i.e. samples) to identify it with confidence must also have polynomial scaling for efficient computability.

Together, these constraints can limit the practicality of implementing certain classes of quantum advantage. One illustrative example relates to the previously mentioned QLSA. In particular, the number of samples required to output the complete solution vector is expected to scale polynomially with *n*. Given the algorithm runs in *O*(polylog(*n*)) operations, fully extracting the solution vector abrogates the superpolynomial speedup [72]. As such, this particular algorithm (and many related ones) are expected to deliver a superpolynomial advantage only for sampling statistics of the solution vector or when calculating some global property of the state. More generally, these output constraints emphasize the importance of choosing an appropriate input encoding in anticipation of the number of classical bits that will be required to output the solution.

## 4. The pursuit of NISQ advantages

### 4.1. A brief overview of variational quantum algorithms

**Figure 2.**
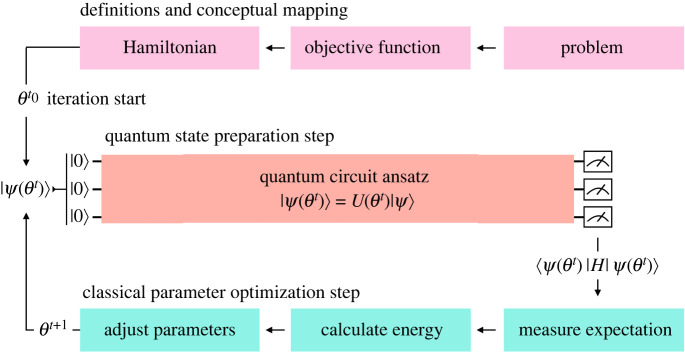
Schematic of a VQA. Development of a VQA begins with the definition of a problem Hamiltonian, *H*, and an appropriate real-valued objective function, L. The problem Hamiltonian is encoded into the quantum device as a unitary operator, $U(\theta )=e^{-iH\theta_{t}}$. In the case of the variational quantum eigensolver (VQE) [136], the objective function is the expectation of the Hamiltonian operator, L(θ)=⟨ψ(θ)|H|ψ(θ)⟩; however, other objective functions are also possible, such as ones based on fidelity [137]. Note here that |*ψ*(*θ*)〉 represents the quantum state parametrized with the real-valued parameter vector, $\theta \in R^{d}$. The VQA then proceeds as follows. First, the qubits of the parametrized quantum circuit are initialized to the fiduciary state |0〉^⊗*n*^ and the parametrized quantum circuit is executed, yielding the initial trial state |*ψ*(*θ*)〉 = *U*(*θ*)|0〉. Next, a measurement on this state is performed to compute the loss of the objective function, L. A classical optimizer (e.g. a simple form of gradient descent uses the update rule $\theta^{t+1}\;:\;=\theta^{t}-\eta \nabla L(\theta^{t})$, where *η* is a hyperparameter for the gradient step size or learning rate and *t* is the time step) then computes updates to the parameter vector, *θ*, to minimize the loss of the objective function. This proceeds iteratively until the system has converged from the initial trial state to a low energy state of |*ψ*_*θ*_〉. In the case of the VQE, this low energy state represents an approximation of and upper bound on the lowest energy state of the Hamiltonian.

Understanding empirical quantum advantages and their computational benchmarking has taken on additional importance with the development of hybrid quantum–classical approaches. Many of these hybrid approaches developed for NISQ devices can be described as variational quantum algorithms (VQAs). In general, a VQA leverages three basic components: (i) a *parametrized quantum circuit* (PQC), (ii) an objective function, and (iii) a classical optimizer^22^ (figure 2). Examples include the variational quantum eigensolver (VQE) [136] and many variational QML algorithms. Crucially, they allow for substantial flexibility in their implementation and application.

To design a VQA, the first step is to define a problem Hamiltonian *H* (i.e. a *Hermitian operator* that, in the context of quantum simulation, corresponds to the kinetic and potential energy of the physical system), and an associated objective function, L. The Hamiltonian is then mapped to an appropriate PQC, which may be composed of both fixed and/or variational single-, two- or multi-qubit gates. This circuit is controlled by a real-valued parameter vector, $\theta \in R^{d}$, which is made to vary by a classical optimizer that seeks to minimize the objective function. These parameters may control a variety of aspects of the quantum circuit, such as quantum gate rotation angles (e.g. *R*_*y*_(*θ*)), whether a layer or unitary in the circuit should execute, or whether execution should halt upon a condition being reached. As an example, a common VQA approach to quantum simulation is to minimize the expectation value of the Hamiltonian L(θ)=⟨ψ(θ)|H|ψ(θ)⟩ with respect to the parametric quantum state |*ψ*(*θ*)〉 prepared by the quantum circuit. Doing so leads to an approximation of the ground state or minimum eigenvalue of *H*, providing an upper bound on the ground state energy of the Hamiltonian in accordance with the *variational principle* [138].

Using this approach, VQAs may solve problems in the near term that would be otherwise infeasible due to the inherent coherence limitations of NISQ devices. Further, by leveraging classical post-processing techniques on the output observables [102,109,139,140], theoretical and experimental evidence suggests that VQAs can be made resistant to errors via error mitigation techniques.

### 4.2. Variational quantum simulation

Quantum simulation is one of the most anticipated applications for quantum computers, particularly in the domains of condensed matter physics, quantum chemistry, material science and structural biology. Within these fields, a core goal is the scalable simulation of large, strongly correlated fermionic systems with an accuracy far superior to available approximate solutions from classical computation. Realizing this goal may precipitate a novel paradigm within these fields where our ability to understand the material world—superconductors, meta materials, chemistries, catalysts, proteins, metabolites and pharmaceutical compounds—is vastly improved. Given the many superpolynomial advantages offered by quantum simulation algorithms, it is plausible that variational quantum simulation (VQS) will yield many practical applications in the near to medium term. These applications may in turn have a significant impact across a broad variety of research fields and industrial sectors.

The first VQS algorithm (and VQA) was the variational quantum eigensolver (VQE) [136]. The VQE was originally conceived to compute quantitative properties of molecular Hamiltonians by leveraging the variational principle [138]. The first problem instance targeted by the VQE was finding the ground state of helium hydride [136]. Since then, various implementations of the VQE approach have been used to simulate molecular binding [141] and dissociation [142,143], dipole moments [144], chemical reactions [145], and other quantum mechanical systems and their quantities. So far, these practical implementations have all focused on simple molecules with relatively few atomic nuclei (i.e. simulations that are classically tractable with greater accuracy).^23^ These molecules include multiple hydrides, such as ones of beryllium (BeH_2_) [146] and sodium, rubidium and potassium (NaH, RbH and KH, respectively) [147]. While these represent promising early results, significant work remains to scale VQS approaches to larger molecules that have biological relevance, such as proteins, enzymes, nucleic acids, and metabolites. In fact, the simulation of such a biomolecule to high accuracy would likely represent an unambiguous demonstration of an experimental quantum advantage.

A number of barriers currently prevent VQS advantages from being realized. Most clear among these are current limitations around the fidelity and size (i.e. number of qubits available) of existing NISQ devices. However, it is expected that this barrier will naturally be addressed as quantum hardware matures. Another relates to the number of measurements required by VQS algorithms to attain chemical accuracy competitive with existing classical simulation techniques [148,149]. Fortunately, substantial recent work suggests that this measurement problem may not be insurmountable [150–153]. A third relates to whether classical optimization algorithms leveraged by VQAs are sufficient to learn the quantum circuit parametrization(s) required to model the physical aspects of large Hamiltonian simulation problems that challenge classical algorithms [154]. While substantial effort has gone into developing quantum aware optimizers [155–164], without this capability the superpolynomial advantages established by existing theoretical quantum simulation algorithms [74–76,83,165,166] (which generally assume FTQC hardware) may not be realized by near-term VQS approaches, which are inherently heuristic.

Nonetheless, it seems unlikely that these barriers will prevent the achievement of practical simulation advantages with VQS algorithms. Significant theoretical [163,167], numerical [168] and experimental evidences [104,145] imply that VQS approaches may be among the best candidates for operational quantum advantages in the near term. In §5.1, we detail a large number of potential applications for quantum simulation and whether they may yield empirical quantum advantages in the near term.

### 4.3. Variational quantum machine learning

Prior to the advent of the VQA paradigm, the field of quantum machine learning (QML) grew rapidly following the publication of the quantum linear systems algorithm (QLSA) [50], which yielded a superpolynomial advantage on a classically easy problem. The QLSA algorithm solves linear systems by matrix inversion, a general technique used by many machine learning algorithms (e.g. to invert a feature covariance matrix). Given the generality of the QLSA, early QML research largely sought to improve upon it [169–172] and leverage it towards the development of other QML algorithms for FTQC hardware. Examples of these include quantum algorithms for support vector machines (SVM) [51], recommendation systems [52], clustering [173], principal components analysis [174] and Gaussian processes [175,176], among many others.

However, following these initial successes several challenges (including the input and output constraints discussed in §3.4) were clarified that, together, implied that the QLSA may not be practical in the near term [72]. Among these is ‘dequantization’ [177], the first example of which occurred in 2018 and involved the development of a classical algorithm for efficient ℓ_2_-norm sampling of approximate matrix products.^24^ Other QML algorithms based on the QLSA were subsequently dequantized, including ones for principal component analysis and supervised clustering [178], SVM [179], low-rank regression [180,181], semi-definite program solving [182] and low-rank Hamiltonian simulation and discriminant analysis [183].

Despite the practical challenges for early QML algorithms, the development of variational approaches has broadened QML into a substantially more diverse field with many algorithms that may deliver practical advantages before FTQC is available. In general, QML approaches can be placed into one of four categories on the basis of the type of data and device being used [184]:

1. A learning algorithm executed on a *classical device* with *classical data*.
2. A learning algorithm executed on a *classical device* with *quantum data*.
3. A learning algorithm executed on a *quantum device* with *classical data*.
4. A learning algorithm executed on a *quantum device* with *quantum data*.

Category **1** represents classical machine learning approaches, including deep learning. In the context of QIS, classical machine learning may find relevance for benchmarking QML algorithms, controlling quantum hardware, and designing quantum experiments [185]. At present, category **2** finds primary application in characterizing the state of quantum systems, studying phase transitions, and high-energy physics. Category **4** may be particularly relevant to domains at the intersection of quantum metrology [186] and QML where quantum data are received as input.^25^ Alternatively, a QML algorithm could be used to post-process quantum data generated by a quantum simulation algorithm, such as the VQE.

Variational QML approaches typically belong to categories **3** and **4**. These algorithms include variational incarnations of the QLSA [187–189], a broad variety of quantum neural networks (QNNs) (e.g. variational autoencoders [190], generative adversarial networks [191,192], continuous variable QNNs [193] and reinforcement learning [194]),^26^ and at least one example of a variational approach to quantum unsupervised learning [195]. For reviews of this area of the QML field, see [196,197].

Among variational QML approaches, quantum kernel estimation (QKE) [132,198,199] and variational quantum classifiers (VQC) [132,133,198] for supervised learning are of particular interest. In the case of QKE, the aim is to leverage a quantum circuit that implements a nonlinear map between the classical features of each class and corresponding quantum states.^27^ The inner product of the two quantum states, corresponding to a quantum kernel function measuring the distance between the data points, is then sampled from the quantum device for each sample pair. This kernel, which may be intractable to compute classically [198,200], can then be input into a classical machine learning algorithm, such as an SVM, for a potential quantum advantage. In this respect, QKE can be viewed as combining both categories **2** and **3** in a single method. A recent demonstration of this approach on a 67 feature astronomy dataset using 17 qubits provided evidence that QKE can yield comparable performance to a classical SVM (in this instance, with a radial basis kernel) on a practical dataset [200]. Further, advantages were recently shown to be possible using these methods on engineered datasets [201]. Whether these advantages can be realized on realistic datasets remains the subject of ongoing research efforts.

Similarly, VQCs aim to learn a nonlinear feature map that embeds the classical features into a higher-dimensional quantum feature space (i.e. a region of the Hilbert space of the quantum system) and maximizes the class separation therein. Doing so allows for the use of a simple linear classifier and can be viewed as a variational analogue to a classical SVM. Crucially, the VQC approach yields a general framework for quantum supervised learning [133]. In particular, VQCs can be viewed as implementing a two step process of (i) embedding classical data into a quantum state and (ii) finding the appropriate measurement basis to maximize the class separation. This approach can be further generalized to allow for other fixed circuits, such as amplitude amplification or phase estimation, following the data embedding phase [202]. A key benefit of VQCs is that the quantum circuit directly outputs a classification, which greatly reduces the number of measurements required relative to QKE.

While many variational QML methods lack analytical bounds on their time complexity (unlike many of the earlier QML algorithms), they may nonetheless offer other forms of quantum advantage. In particular, theoretical and empirical evidence exists for improvements in generalization error [137,203–205], trainability (i.e. with certain constructions, favourable training landscapes with fewer barren plateaus and narrow gorges [137,203,206–209]), and sample complexity [210–212]. It is plausible that these types of advantages may lead to novel machine learning applications in biology and medicine. However, recent work has also made clear that the bar for achieving these advantages may be high given the ability of data to empower classical machine learning algorithms [201].

Explorations of variational QML approaches for biology and medicine are only now getting under way. They include proof of principle implementations of protein folding with a hybrid deep learning approach leveraging quantum walks on a gate-based superconducting device [213] and the diagnosis of breast cancer from legacy clinical data via QKE [214]. As larger, more flexible quantum devices are made available, further growth of research into applications of variational QML is expected.

### 4.4. Quantum approximate optimization algorithms

The quantum approximate optimization algorithm (QAOA) is a hybrid quantum–classical algorithm for targeting optimization problems, such as MaxCut [215]. Following its original publication, the algorithm was generalized into a broader framework, called the quantum alternating operator ansatz (retaining the original acronym) [216,217]. This generalization allows for more expressive constructions capable of addressing a wider range of problems. Below, we briefly review the original QAOA approach; we refer to Hadfield *et al.* [216] for more details including extensions to the algorithm.

The original QAOA leverages two Hamiltonians: a phase (i.e. problem) Hamiltonian, *H*_*P*_|*y*〉 = *f*(*y*)|*y*〉, and a mixing Hamiltonian, $H_{M}=\sum_{\;j=1}^{n}X_{j}$. *H*_*P*_ encodes the cost function which operates on an *n* qubit computational basis state and *H*_*M*_ is comprised of a Pauli-*X* operator for each qubit. Application of the unitary operators generated by *H*_*P*_ and *H*_*M*_ to the initial state is then alternated for *p* rounds. Here, *p* represents a crucial parameter of the QAOA algorithm, defining the length of the quantum circuit and the number of its parameters. There are 2*p* parameters of the form *γ*_1_, *β*_1_, *γ*_2_, *β*_2_, …*γ*_*p*_, *β*_*p*_, which control how many iterations of the alternating Hamiltonian operators are applied. The QAOA circuit thus prepares the parametrized quantum state,

4.1 $$|\beta ,\gamma \rangle =e^{-i\beta_{P}H_{M}}\;e^{-i\gamma_{P}H_{P}}\;\ldots \;e^{-i\beta_{1}H_{M}}\;e^{-i\gamma_{1}H_{P}}|s\rangle ,$$

which is an unbalanced superposition of computational basis states (typically, |*s*〉 is initialized as a balanced superposition). By measuring all qubits in the computational basis, a candidate solution, *y*, is obtained with probability |〈*y*|*β*, *γ*〉|^2^. By repeated sampling of the state preparation and measurement steps, the expected value of the cost function over the returned samples can be computed as 〈*f*〉 = 〈*β*, *γ*|*H*_*P*_|*β*, *γ*〉 (i.e. using the phase Hamiltonian).

In many respects, the QAOA algorithm and its generalization are similar to the VQA framework described in §4.1. However, unlike VQAs, QAOA algorithms are less flexible in the problems they can address. This is due to (i) limitations on the construction of the problem Hamiltonian and (ii) the singular *p* hyperparameter, which defines how many applications of the problem and mixing Hamiltonians should occur. Together, these characteristics may limit the expressivity of QAOA algorithms and, in turn, their ability to yield a practical quantum advantage. Despite these potential challenges, the QAOA algorithm may have some benefits over other quantum algorithm approaches due to its relatively short circuit depth,^28^ which aligns with the limited coherence of NISQ devices.

With respect to quantum advantages, recent numerical simulations have estimated that an experimental advantage would require hundreds of noisy qubits [218,219]. Such a NISQ device appears plausible in the near term [220] and potential advantages using the QAOA framework are being explored on at least one state-of-the-art superconducting platform [221]. Further, the QAOA framework may also broaden the scope of possible quantum advantages to improved approximation ratios on hard optimization problems.^29^ Yet, the exact problems for which practical advantages may exist are at present unclear. Relevant to biology, we know of two recent examples of QAOA-based proof of principles. The first applied QAOA to the task of protein folding in a lattice-based model [222], while another used QAOA to develop an overlap-layout-consensus approach for *de novo* genomic assembly [223].

To address the gap around the existence of problems where a quantum advantage may be possible, recent work has involved the development of a framework for searching for QAOA-based advantages [224] and a classical machine learning approach for identifying problems that may offer an advantage [225]. These general approaches, designed to aid in the targeting of problems that may yield a quantum advantage, remain relatively unexplored. Other work has focused on the characteristics of quantum information and the challenges they present to optimization in the quantum regime, which has led to insights around the type of structure needed for quantum advantages in low-depth circuits [226]. As is the case with many quantum algorithms, it is too early to say whether QAOA approaches will provide quantum advantages in practice.

### 4.5. Quantum annealing

*Quantum annealing* (QA) devices provide an alternative approach to quantum computing with specialized NISQ hardware based on classical simulated annealing that may yield quantum advantages in quantum simulation and optimization. Unlike the many gate-based NISQ devices, quantum annealers provide a form of analogue computation based on the *adiabatic model of quantum computation* [227,228].

Existing commercial quantum annealers are qualitatively different from programmable gate-based devices.^30^ Most notably, their higher qubit counts (now of the order of 10^3^) and dense qubit connectivity maps allow for relatively large and complex inputs. However, despite these benefits, a number of drawbacks also exist. These include (i) a non-universal model of computation,^31^ (ii) their analogue nature, which is expected to limit the ability to perform error correction (although error mitigation appears possible), and (iii) the development of a quantum-inspired classical algorithm, simulated quantum annealing (SQA), capable of efficiently simulating quantum tunnelling (see [229] for an overview).^32^ With respect to the latter, however, it is notable that while quantum tunnelling may be efficiently simulatable by SQA, experimental work has demonstrated performance consistent with empirical advantages on artificial problem instances with characteristics thought to mirror large-scale instances of optimization problems [230,231].

Given their early development, large qubit counts and robust connectivity maps, multiple proof of principle demonstrations targeting bioinformatics and computational biology applications have been developed using quantum annealers. These include ranking and classification of transcription factor binding affinities [232], the discovery of biological pathways in cancer from gene-patient mutation data [233], cancer subtyping [234], the prediction of amino acid side chains and conformations that stabilize a fixed protein backbone (a key procedure in protein design) [235], various approaches to protein folding [236–240] and two recent approaches for *de novo* assembly of genomes [223,241].

However, despite many promising proof of principle demonstrations ([242] represents a notable example), no clear practical quantum advantage has been shown. The degree of advantage possible by QA algorithms also remains unclear and this ambiguity arguably extends over the long term. The primary factors contributing to this uncertainty are the aforementioned non-universal model of computation and the lack of clarity around the possibility for error correction. Thus, while QA approaches to optimization may yet bear fruit in the NISQ era [243], a number of barriers remain to be addressed [226].

## 5. Future prospects in biology and medicine

In this section, we describe a broad variety of potential applications for quantum algorithms. Our aim is to highlight both the breadth of existing quantum algorithms and the types of problems in biology and medicine that they may address. We leverage the quantum advantage framework described in §3 and note when a specific application may admit an empirical quantum advantage in the near or medium term (summarized in table 3). While some of the applications described are not expected to be feasible in the near term—indeed, in some cases, even an FTQC may not be the most appropriate tool for the target problem—it is our intent for the breadth of potential applications and research directions covered to be valuable to an interdisciplinary audience. As such, when possible, we have sought to provide (i) quantum scientists with relevant details and references to develop targeted quantum algorithms for applications in biology and medicine and (ii) domain computationalists with information on quantum algorithms relevant to applications in biology and medicine and their prospects for operational quantum advantages in the near or medium term.

**Table 3.** Selected quantum proof of principles relevant to biology and medicine. A variety of quantum approaches to addressing computational problems in biology and medicine exist, some of which have been experimentally demonstrated. Expected empirical advantages vary greatly. Among the most promising near-term applications are ones that leverage quantum simulation and quantum machine learning techniques, such as quantum neural networks.

target application	experimental demonstration	hardware device	algorithm type	classical complexity	expected advantage
protein folding and conformation simulation [236–240]	yes	quantum annealer	quantum annealing	polynomial; heuristic approximation	unknown, up to polynomial
molecular docking simulation [244]	no	Gaussian Boson sampler	sampling	super-polynomial	unknown; up to super-polynomial
*de novo* assembly [223,241]	yes	quantum annealer; universal gate-based quantum device	quantum annealing, optimization	polynomial; heuristic approximation	unknown, up to polynomial
sequence alignment [67–69,245,246]	no	universal gate-based quantum device	optimization	polynomial; heuristic approximation	polynomial
sequence matching [71,247,248]	no	universal gate-based quantum device	QML; search	polynomial	up to super-polynomial
inference of phylogenetic trees [70]	no	universal gate-based quantum device	optimization	super-polynomial	polynomial
inference of biological networks [233,249,250]	yes	quantum annealer	optimization	polynomial and super-polynomial	polynomial
transcription factor binding analysis [232]	yes	quantum annealer	optimization	polynomial; heuristic approximation	unknown, up to polynomial
neural networks [137,193,203,204,248,251–255]	yes	universal gate-based quantum device	QML	polynomial and super-polynomial (e.g. Boltzmann machine)	polynomial, problem specific, varies by measure (e.g. [203,256])

### 5.1. Simulating quantum physics

Simulating microscopic properties and processes at the atomic level [257] is a key area of computational biology research. These tasks often require quantum mechanical simulations, which are classically intractable for all but the smallest quantum systems. These inherent limitations mean that most classical approaches are approximations and often provide a mostly qualitative understanding. By contrast, many of these same quantum mechanical simulations are a natural task for quantum computers. Beyond the NISQ era, it is expected that simulations of large quantum systems may be used to predict biochemical properties and behaviours that are not efficiently computable with classical devices [168,258,259].

In this section, we begin with an overview of applications in or relevant to biology and medicine that may benefit from quantum simulation approaches. We then provide a brief summary of three quantum algorithms central to quantum simulation. Finally, we conclude with a discussion of the potential for empirical quantum advantages in the near term.

#### 5.1.1. Ground states, binding energies and reactions

Calculating the energy of a molecular system is a ubiquitous task in computational chemistry [260]. An important example (which calculates ground states as one of several subroutines) is protein–ligand docking, where the goal is to calculate the binding energy of a small molecule (e.g. a drug) to a target site on a protein or other macromolecular structure (e.g. a receptor domain) [261]. Though this problem type is often approximated with classical mechanics, future quantum computers may provide highly accurate predictions for docking. Similar types of simulations may find use as subroutines for calculating protein–protein interactions and small-molecule properties, like the solubility of drug molecules [262,263]. In addition, calculating the ground state along different nuclear positions yields reaction coordinates, which are essential for understanding both reactivity *in vivo* and drug synthesis mechanisms [264].

#### 5.1.2. Molecular dynamics

Molecular dynamics (MD) simulations involve propagating the equations of motion of a microscopic system, such as a complex containing proteins or DNA [265]. In addition to understanding qualitative mechanisms, a core goal is often to calculate quantities, such as diffusion rates [266] and Gibbs free energies [267]. To do this, MD often uses parametrized force fields and Newtonian dynamics, although one can accurately treat nuclear quantum effects (such as tunnelling and zero-point energy) via path-integral MD methods [268], and electronic quantum effects using, for example, Car–Parrinello MD [269]. In principle, quantum simulation approaches may be used for both time propagation (using quantum algorithms that speed up classical ordinary differential equations) [270,271] and electronic structure calculations performed at each time step [272].

#### 5.1.3. Excited states

Though excited electronic and vibrational [273,274] states are not usually a primary focus in biological processes, they are important for probing microscopic states using spectroscopy [275,276]. Green fluorescent protein (GFP), for example, is a commonly used marker that allows one to study the expression, localization and activity of proteins in cells via microscopy [277,278]. Other artificial dyes and markers have also been used to study dynamic processes, such as diffusion [279] and DNA unravelling [280]. In principle, the ability to accurately compute excited states could lead to effective screening methods to aid the development of novel fluorescent proteins and dyes that emit or absorb highly specific wavelengths, have narrower emission/absorption bands, or exhibit higher quantum efficiency. These dye markers may also be probed by a variety of spectroscopy methods, such as absorption, emission and Raman spectroscopy^33^ [275,276,278]. In addition, time-dependent femtosecond spectroscopy is often necessary when studying certain biomolecular processes and the ability to model femtosecond excited-state behaviour could allow for more accurate interpretation of certain experiments [281]. Finally, other excited-state processes inherent to biological systems exist that may benefit from quantum approaches, such as photosynthesis [281] and modelling simple tissue degradation via ultraviolet light [282].

#### 5.1.4. Electronic dynamics

Deeper understanding of some biologically relevant processes might be achieved from the simulation of electron dynamics.^34^ Cases where one may need to directly simulate the dynamics of electrons include enzymatically driven reactions such as nitrogen fixation [258], biomolecular signalling [283], biological processes involving radical reactions [284,285], component processes of neurons and synapses [286], photosynthetic processes [281] and the interpretation of electronic behaviour in femtosecond spectroscopy experiments, as mentioned above. Some related fundamental phenomena might also be better understood via direct simulation. One notable example is proton-coupled electron transfer (PCET) [287], which is ubiquitous but only partially understood. The applications for knowledge generated from simulations of electronic dynamics vary widely. One future possibility could be the design of novel enzymes for the development of more sensitive and specific diagnostic assays or novel therapeutics [288].

#### 5.1.5. Hybrid quantum–classical models

When modelling a large biomolecular system, researchers sometimes implement a model that uses different approximations for different portions of the system. For example, one might use Newtonian molecular dynamics for the majority of a protein, but perform electronic structure calculations for the protein’s reaction site. Another example is to use density functional theory (DFT) [289] as a lower accuracy method and dynamical mean-field theory (DMFT) [290] as a higher accuracy method for a subsystem of interest [291]. Currently, classical examples of such multi-layered approaches exist, such as the ONIOM method (own *N*-layer integrated molecular orbital molecular mechanics) [292]. In principle, these existing classical approaches could be modified to run a classically intractable portion on a quantum computer, leaving the rest to run on a classical computer. Already, work on quantum algorithms in this direction is being pursued [291,293].

#### 5.1.6. Quantum algorithms for quantum simulation

Arguably, there are three broad quantum computational methods used when studying quantum physical systems: time propagation [37,74], quantum phase estimation (QPE) [294,295] and the variational quantum eigensolver (VQE) [136,163,296]. We describe the basic versions of these algorithms below.

#### 5.1.7. Time propagation

When one is interested in propagating the dynamics of a system, the goal is to approximate the time-propagation operator

5.1 $$|\psi_{0}(t)\rangle =U(t)|\psi_{0}\rangle =\exp(-itH)|\psi_{0}\rangle ,$$

where *H* is the Hamiltonian describing the system of interest. For near-term hardware, this is most easily performed using the low-order Suzuki–Trotter decompositions [297,298], though asymptotically more efficient algorithms also exist for fault-tolerant devices [299–305]. Note also that QPE, discussed next, uses time propagation as a subroutine.

#### 5.1.8. Phase estimation

For an arbitrary Hamiltonian, the QPE algorithm outputs the phase ($\;e^{-iE_{i}\tau }$) of the eigenenergy *E*_*i*_ for arbitrary *τ*, given the input of an eigenvector |*ψ*_*i*_〉,

5.2 $$|\psi_{i}\rangle |0\rangle \overset{\mathrm{QPE}}{\to }|\psi_{i}\rangle |\;e^{-iE_{i}\tau }\rangle .$$

When the input is a mix of eigenvalues, the probability of measuring a particular eigenvalue (eigenphase) is proportional to its overlap-squared. Assuming one has an FTQC and a method for preparing an eigenstate of interest (for example a molecular ground state), the QPE algorithm can be used to output the eigenenergy. One can readily determine the eigenvalue *E*_*i*_ from $e^{-iE_{i}\tau }$, whose precision depends on the number of additional qubits in the second quantum register.

#### 5.1.9. Variational quantum eigensolver

For early generations of quantum hardware, it is likely that approaches based on the VQE will be the only viable option.^35^ In this method, the goal is to minimize the function

5.3 $$\min_{\vec{\theta }}\langle \psi (\theta )|H_{\mathrm{sim}}|\psi (\theta )\rangle$$

by varying the parameters θ. These parameters determine the behaviour of the quantum circuit, which prepares the quantum state |ψ(θ)⟩. Usually these parameters simply control rotation angles for one- and two-qubit gates. A recent review of the VQE [296] discusses many of the extensions to the algorithm that have been proposed.

In many cases, these algorithms—time propagation, QPE and VQE—are extensively modified. For example, a variety of strategies to enhance their capabilities have been leveraged in experiments. Among these strategies are error mitigation [97–101,103–108], post-processing to improve accuracy [306,307], approaches to reducing the number of quantum circuit evaluations [150,308,309] and approaches to dynamically modify the quantum circuits [143]. It is anticipated that these types of practical enhancements, among others, will be crucial to realizing many empirical quantum advantages in the near term both within and outside the space of quantum simulation problems.

#### 5.1.10. Prospects for quantum simulation

Quantum simulation offers some of the strongest prospects for practical quantum advantages. Example applications include finding ground [146,147] and excited states [163,310] of electronic degrees of freedom, vibrational degrees of freedom [273,274] and more complex degrees of freedom [82,311–313], or dispersion interaction between drug molecules and proteins [314]. VQEs in particular have the strong possibility of near-term advantages as they scale. In this respect, one promising direction is divide-and-conquer approaches [315,316], which combine multiple VQEs by hierarchical methods to simulate molecules that would otherwise be too large to input into current NISQ hardware. Provided these approaches prove practical, it is possible that simulating larger, biologically relevant molecules—proteins, nucleic acids, drugs and metabolites—will be feasible in the near term.

Further, potential also exists for other hybrid quantum–classical models. Already, quantum algorithms for embedding models have been developed [291,293]. Similar algorithms may allow for the treatment of a subsystem (such as a protein active site) with the quantum computer while the rest of the system (such as the solvent and protein) is simulated with a classical computer. Like the VQE and short-duration quantum dynamics, these hybrid approaches may yield empirical quantum advantages in the near to medium term.

In principle, quantum simulation approaches may also be used for both time propagation and electronic structure calculations, which could be performed at each time step. It is possible that short-time quantum dynamics simulations (using quantum algorithms that speed up classical ordinary differential equations, discussed in the next section) [270,271] on near-term devices may find limited use in elucidating reaction mechanisms and approximating free energies that remain classically intractable.

Over the long term, it is possible that quantum simulations may expand to include QPE on FTQC devices. These methods would allow for the precise quantification of biochemical properties and behaviours far beyond the capabilities of classical HPC systems [168,258,259]. Further, it is also possible that accurate, long duration *ab initio* MD simulations may also be attainable with these methods over the long term.

### 5.2. Simulating classical physics

Simulations of biological processes governed by classical physics often require searching over a parameter space to optimize a set of classical variables, for which search, optimization and machine learning algorithms may be used. In addition, the simulation of Newtonian physics and other non-quantum processes is widely used in biological research. For these simulations, ordinary (ODEs) and partial differential equations (PDEs) are particularly important and have broad applications—from simulating fluid and tissue mechanics to molecular dynamics. Below, we first summarize this application space, briefly review relevant quantum algorithms, and then consider the prospects for empirical quantum advantages.

#### 5.2.1. Conformation search

It is often necessary to search a large conformational space in order to find a global or near-global optimum [317–319]. Such a search is usually performed over a domain of classical variables (e.g. Cartesian atomic coordinates), and may be done in concert with a quantum mechanical method for calculating the energy at each given conformation (as noted above). An important example of conformation search in biology is protein folding [317], but large conformation spaces are also encountered when studying other biomolecules, such as RNA [320], determining a drug’s molecular crystal structure [321], or identifying pathways in complex reaction mechanisms [322].

#### 5.2.2. Fluid mechanics

Many biological processes are governed by fluid mechanics, requiring the simulation of Navier–Stokes equations [323]. Relevant macroscopic processes in this area include the simulation of cardiovascular blood flow [324] and air flow in lungs [325], as well as some aspects of gastroenterology [326]. On a smaller scale, one may want to simulate highly viscous flow inside or around microbes [327], or capillary flow [328]. The latter is especially relevant to angiogenesis [329] (a hallmark of cancer [20,21]) and understanding tumour formation and drug permeability [330]. Further, fluid simulations may be used to model designs for chemical reactors and bioreactors, which are often critical components in drug [331] or complex tissue [332] manufacture.

#### 5.2.3. Non-fluidic continuum mechanics

Macroscopic modelling is important not just for fluids but also for solid or semi-solid continuum materials. In this context, the finite-element method and related approaches are often used [333,334]. While applications of these methods include the modelling of macroscopic tissues, such as muscle [335] and bone [336], they have also been applied to the nanoscale, including in simulations of the cytoskeleton [337].

#### 5.2.4. Classical electrodynamics

Classical electrodynamics can ultimately be described by differential equations, too. The design of medical devices is one area where this type of simulation may be useful. Examples may include the modelling of MRI designs [338] or, perhaps, medical devices that interact with lasers. Another area of application is the design of classical optical devices [339], which may be used in biological research [340].

#### 5.2.5. Systems modelling and dynamics

There are many ubiquitous classical modelling approaches apart from those that directly use Newtonian physics. A particularly relevant example exists in complex population models, which are essential in fields such as epidemiology [341,342]. Others include detailed simulations of entire cells [343], organs [344] or groups of organisms [345,346].

#### 5.2.6. Quantum algorithms for classical simulation

A number of targeted quantum algorithms have been proposed for finding low-energy conformations and searching through candidate molecules, many of which have been specifically developed for protein folding [317,319,347]. More generally, amplitude amplification [46] may be used to explore conformation spaces over classical variables with a quadratic advantage. Additionally, theoretical quantum advantages have also been shown for other optimization-related subroutines, such as escaping saddle points in optimization landscapes [348]. Adjacent to optimization and search, QML algorithms may also be applied. Already, examples exist, such as one for leveraging quantum deep learning to search the chemical space [349,350]. It is plausible that empirical advantages with these methods may be achievable in the near term given their hybrid quantum–classical structure. Finally, the past few years have also seen progress in quantum algorithms for solving classical differential equations, either for general cases [270,271,351–353] or specific applications, like the finite-element method [354,355] or Navier–Stokes [356,357]. Importantly, among these quantum algorithms are ones for solving the more difficult cases of non-homogeneous and nonlinear PDEs [271,353].

#### 5.2.7. Prospects for classical simulation

With respect to search over conformation spaces, it is possible that empirical quantum advantages may be achievable. However, recent work has indicated that general approaches offering quadratic advantages, like amplitude amplification, may be of limited value on their own, even in the FTQC regime [41]. For this reason, it is expected that the integration of domain knowledge and additional quantum subroutines will be key to achieving any future advantages. For QML, and hybrid quantum–classical algorithms in particular, further exploration of near-term compatible methods to improve simulations of classical physics is merited. Finally, the two primary aspects of fluid simulations that lead to simulation difficulty are, arguably, system size and turbulent flow [358]. While it is unclear whether turbulence may be addressed efficiently with quantum approaches, quantum algorithms for differential equations may allow for reductions in complexity with respect to system size [271,353], which could lead to superpolynomial advantages in some cases. However, a caveat also exists with known quantum algorithms for differential equations given that they are affected by the input and output problems described in §3.4. For this reason, further research is required to understand when empirical quantum advantages for these applications may become feasible.

### 5.3. Bioinformatics

Optimization is central to many bioinformatics tasks, such as sequence alignment, *de novo* assembly, and phylogenetic tree inference. At their core, classical algorithms for these problems often use subroutines for matching substrings, constructing and traversing string graphs, and sampling and counting *k*-mers (i.e. substrings of biological sequences). Here, we describe the basic constructions of these problems and summarize relevant quantum algorithms.

#### 5.3.1. Sequence alignment

Sequence alignment is a computational primitive of bioinformatics tasks. The heavy integration of sequence alignment algorithms into bioinformatics software has led to diverse applications—from the *de novo* assembly of whole genomes [359], to the discovery of quantitative trait loci linked to disease phenotypes [360] and identification and analysis of driver mutations in cancer [361]. A classic formulation for identifying the global optimum alignment of two sequences involves finding the lowest weight path through an *n* × *m* dynamic programming matrix, where *n* and *m* are the lengths of the sequences being compared (it is often the case that *n* = *m*) [362]. In practice, an approximate solution is typically constructed by a greedy heuristic using a biologically informed scoring function. Examples of scoring functions include sum-of-pairs, weighted sum-of-pairs and minimum entropy (each of which imply certain biological assumptions) [363]. For example, with a weighted sum-of-pairs, one may assign different scores to DNA base matches, mismatches, substitutions, insertions and deletions (the scoring system may also be used to control whether the output alignment is global [362] or local [364]). Alternatively, for proteins, a scoring matrix may be used where each cell represents the likelihood that the amino acid in the row will be replaced by the amino acid in the column [365]. This likelihood may be determined empirically by a statistical analysis of a large protein sequence database or on the basis of chemical properties of the amino acids (e.g. polar or non-polar, hydrophilic or hydrophobic). While pairwise alignment has polynomial complexity, the generalization to multiple sequence alignment (MSA) with sum-of-pairs scoring is known to be NP-hard [366–368]. Other heuristic approaches to sequence alignment also exist, such as progressive alignment [369]. For more on MSA algorithms and their broad applications, see this recent review [370].

#### 5.3.2. *De novo* assembly

*De novo* assembly refers to the process of assembling a reference genome—a foundational resource for many bioinformatics analyses—from a large set of overlapping reads of the genome. Often these are short reads (of the order of 10^3^ base pairs long with error rates of the order of 10^−5^ [371]). However, more recent long read sequencing technologies (typically of the order of ≥10^5^ base pairs with error rates of the order of 10^−3^ [372]) can also be used to aid in the scaffolding of the genome using a hybrid approach [359],^36^ which was recently used to great effect during the development of a novel human reference genome [373]. Modern software packages typically leverage one of two approaches: (i) overlap-layout-consensus (OLC), which involves the construction of a string overlap graph and is reducible to the NP-complete Hamiltonian path problem (a greedy approximation heuristic is used) and (ii) a *k*-mer graph approach, which involves the construction of a de Bruijn graph and is reducible to the Eulerian path problem, which admits a polynomial time algorithm. In practice, achieving high-quality, biologically plausible assemblies is non-trivial and subject to many challenges due to both genome structures, such as homopolymeric and repetitive regions, and the introduction of errors into sequencing data from library preparation, systematic platform error and low coverage regions [374].

#### 5.3.3. Phylogenetic tree inference

Phylogenetic tree inference is the process of inferring the evolutionary relationships between multiple genome sequences.^37^ Typically, inference of a gene-based *phylogeny* involves combining (i) a multiple sequence alignment of the genomes in question, (ii) an evolutionary dynamics model accounting for the types of evolutionary processes occurring between sequences, and (iii) a tree’s topology, where branch lengths represent the distance (e.g. Hamming or Levenshtein distance) between two sequences. The evolutionary dynamics model may be as simple as a continuous-time Markov model sampling from a table of specific events with empirically estimated transition probabilities (e.g. a base substitution *T* > *A*; in evolutionary biology, the events and their probabilities may be highly specific to a species or genus). Alternatively, more complex Bayesian methods and mixture models may be used where the associated probabilities for events may vary for different sequence regions. The topology of a phylogenetic tree may be initialized randomly from the MSA and is often inferred by hierarchical clustering with a maximum-likelihood estimator (MLE) [375]. In practice, phylogenetic tree methods are central to evolutionary biology [376] and understanding the evolutionary dynamics of clonal populations in cancer [377] (among a multitude of other applications), the latter of which has significant clinical relevance to the targeting of precision therapeutics and characterization of treatment resistance.

#### 5.3.4. Emerging application areas

Many emerging application areas in bioinformatics exist that may represent interesting targets for quantum algorithm development. Examples of application areas include (i) the inference of topologically associating domains (TADs; interacting regions of chromosomes governed by the three-dimensional bundling structure of chromatin in cell nuclei), which are crucial to our understanding of epigenetic mechanisms [378], (ii) single-cell multiomics, a set of novel methods for generating multimodal data from single-cell sequencing assays, which allow for simultaneous measurement of a cell’s state across the biological layers (e.g. genomic, transcriptomic and proteomic) [379], and (iii) improving the modelling and inference of biological networks (e.g. interaction networks for genes, transcripts or proteins). With respect to the latter, this may include the alignment of multimodal networks [380–382], which can be used for predicting the associations between biological and disease processes [381,382], and the modelling of gene regulator networks using complex-valued ODEs [383], which may be especially well suited to quantum information. Given the breadth of the bioinformatics space, these applications represent a very small subset of the potential emerging application space for quantum algorithm development.

#### 5.3.5. Prospects for bioinformatics

A small number of quantum algorithms for problems in bioinformatics have been proposed (table 3). These include theoretical algorithms developed for FTQC devices that target NP-hard problems, such as sequence alignment [67–69] and the inference of phylogenetic trees [70], which leverage amplitude amplification and quantum walks [384]. To be made practical, these theoretical quantum algorithms are expected to require both significant refinement and effort in translation. In the near term, these refinements could include (i) recasting them for NISQ devices using the VQA, QAOA or QA frameworks and (ii) integrating greater biological context. Already, examples of this type of work exist for *de novo* assembly [223,241], sequence alignment [245] and the inference of biological networks [249,250]. Over the long term, operational advantages may be pursued by optimizing near-term approaches and integrating fast quantum algorithm subroutines where possible. Known quantum algorithms that may be relevant to this work include ones for backtracking [65], dynamic programming [47,48], operating on strings [71,246,247] and differential equations [270,271,351–353].

Taking these measures into account, operational advantages for these problems may nonetheless remain among the most difficult to achieve. This is partly due to the factors discussed in §3.4. Other barriers to quantum advantages include (i) the sophistication of existing classical heuristic algorithms and the inherent parallelism of many of the problems they solve, (ii) the scale of both existing classical hardware and practical problem instances within the context of contemporary research [385], (iii) the broad institutional support and incumbent advantage benefiting existing classical approaches (including extensive clinical validation in the medical setting), and (iv) the likely precondition of FTQC to realize polynomial advantages based on amplitude amplification in practice [41]. Thus, while current research in this direction shows long-term promise and should be explored further, many of these quantum advantages appear unlikely to be practical in the near term.

### 5.4. Quantum machine learning

Many of the quantum advantages associated with near-term variational QML algorithms relate to model capacity, expressivity and sample efficiency. In particular, variational QML algorithms may yield reductions in the number of required trainable parameters [214], generalization error [137,203–205], the number of examples required to learn a model [199,212] and improvements in training landscapes [137,199,203,207,208,252]. Evidence supporting one or more of these advantages has been found in both theoretical models and proof of principle implementations of quantum neural networks (QNNs) [137,203,204,207] and quantum kernel methods (QKMs) [199,201,205]. It is notable that QNNs in particular are closely related to VQAs leveraging gradient-based classical optimizers (indeed, they often share overlapping definitions in the literature, as briefly noted in 2019 [193]) [202,386,387]. Given the breadth of applications for machine learning approaches in biology, we focus our discussion below on these types of advantages and their potential applications in lieu of specific methods.

#### 5.4.1. Improvements to training landscapes

Improvements to training landscapes refer to the reduction or removal of barren plateaus and narrow gorges in the landscape of the objective function of a gradient-based learning algorithm. These improvements may stem from the unitary property of (many) quantum circuits, which inherently maintains the length of the input feature vector throughout the computation [214] provided an appropriate input encoding is used. This bears similarity to many classical approaches used to improve and stabilize training landscapes in practice, such as batch normalization [388] and self-normalizing neural networks [389]. While improved training landscapes may result in more rapid convergence, it is unclear whether this type of advantage alone can be made practical (e.g. by allowing for a model to be trained that would be ‘untrainable’ by classical means). Fortunately, improvements in training landscapes have been seen to co-occur with reductions in generalization error [203,390].

#### 5.4.2. Reductions in generalization error

Generalization error measures the ability of a machine learning model to maintain similar performance (i.e. ‘generalizability’) on unseen data.^38^ Reductions in generalization error may yield advantages in the accuracy and flexibility of trained machine learning models. Advantages in generalization error are dependent on a variety of factors, including the encoding used (with basis encoding performing particularly poorly [391]) and the availability of data sufficient to train a comparable classical model [201]. While there is substantial evidence supporting reductions in generalization error [137,203–205], evidence of poor generalization performance under certain constructions also exists (e.g. [392]). This may be partly attributable to shallower quantum circuits providing better utility bounds than deeper circuits [393], which contrasts with classical neural network intuition where increased layer depth is associated with an exponential increase in model expressiveness [394]. With respect to applications, much like improvements in training landscapes, reductions in generalization error (despite having broad relevance) may alone be insufficient to provide a practical quantum advantage in the near term.

#### 5.4.3. Reductions in sample complexity

Reductions in sample complexity may allow for the learning of robust machine learning models from fewer examples. Intuitively, sample complexity (and generalization error) advantages may arise when quantum entanglement enables the modelling of classically intractable correlative structures. If such sample complexity advantages are achievable with classical data, they will likely be problem instance specific [201], highly dependent on the distribution of the input data [199,201] and are unlikely to be superpolynomial [211,395]. Nonetheless, polynomial [210–212] or even sub-linear reductions in the number of examples required to build a classifier could provide significant operational advantages

The source of these operational advantages can be viewed as the result of the typically high cost of sample generation or acquisition in biological research and clinical contexts. This cost can be due to a variety of factors, including wet laboratory protocol duration, procedural invasiveness, financial cost or low disease incidence. Some emblematic examples of costly data acquisition in the clinical context include the sampling of bone marrow in leukaemias and time-consuming medical imaging of patients with rare neurological diseases.

This significant cost in the acquisition of data contrasts with typical hardware measures of time and space resources, such as clock cycles, gates, (qu)bits and queries, which are often (or expected to be) very cheap due to their high frequency and scalability.^39^ Indeed, where a quadratic reduction in the number of function queries (e.g. from *n* = 10^6^ to $\sqrt{n}=10^{3}$) may lead to only millisecond differences in compute time, a similar reduction in the numbers of samples could save months or years in biological sample collection and processing time (to say nothing of the economic considerations). Further, by providing a potentially large improvement in operational outcomes, sample complexity advantages over classical data distributions may also exhibit a resiliency to improvements in classical hardware. For these reasons, the discovery of structures within data for which quantum computers may offer even small reductions in sample complexity could have substantial relevance to the development of QML approaches for many prediction and inference problems in biology and medicine.

#### 5.4.4. Privacy advantages

Since the earliest days of the QIS field, the inherently private nature of quantum information has been a subject of significant interest [396]. More recently, theoretical work at the intersection of quantum information and differential privacy has been explored [393,397–400], with potential applications in the healthcare setting [401]. While research in this area is in the very early stages, the potential for differentially private learning on large, open healthcare data resources presents an opportunity for classical machine learning techniques and may be further empowered by quantum advantages in differentially private learning and data sharing over the long term. For an example of one classical approach, see [402].

#### 5.4.5. Prospects for quantum machine learning

Variational quantum machine learning (QML) is expected to provide a methodological toolbox with significant relevance to a wide range of biological research and clinical applications. Substantial numerical and theoretical evidence now points towards a variety of strengths related to variational QML algorithms on NISQ hardware. These include robustness in the presence of device, parameter, feature and label noise [137,214,403,404]. It is possible that the breadth of applications may be similar to deep learning, a set of highly flexible methodological tools for generative and predictive modelling now widely used in the field [405–407].

With respect to quantum advantages, further experimental work is necessary to assess whether the potential advantages in variational QML discussed above can yield operational advantages. Sample complexity advantages in particular could have a great impact.^40^ Indeed, if even small polynomial reductions can be demonstrated for data types common in biological and clinical research, they may find important applications where examples are rare (e.g. due to disease incidence) or sample acquisition is expensive, invasive, or difficult. Examples that fit this criterion include the diagnosis and prognosis of rare phenotypes [408,409], the identification of adverse multi-drug reactions from EHR data [410,411], and the diagnosis of cancers [412] and their subtyping on the basis of clinical outcomes, such as drug sensitivity [413] and disease prognosis [414]. Altogether, while practical applications of QML advantages remain largely theoretical, their potential to address existing domain constraints provides ample motivation for further research into variational QML approaches.

### 5.5. Quantum data structures

In bioinformatics and computational biology, non-traditional data structures have long been leveraged by classical algorithms to great effect. For instance, a number of state-of-the-art algorithms for error correcting sequencing data [415] leverage Bloom filters [416], a probabilistic data structure related to hash tables. The core benefit of a Bloom filter comes from its ability to trade a low probability of false positive lookups for significant savings in memory—a common constraint in large bioinformatics pipelines. In a similar vein, the full-text minute space (FM)-index data structure [417] is leveraged (in conjunction with the Burrows–Wheeler transform) by sequence aligners such as Bowtie [418], the BWA family of aligners [419–421] and more recent graph reference genome aligners [422,423]. Like Bloom filters, FM-indexes offer rapid querying and significant memory efficiency.

It is conceivable that the inherently probabilistic nature of quantum computers and novel data input modalities offered by quantum information, such as angle and phase encoding, could lead to the development of similarly useful quantum data structures and abstractions in the FTQC regime. QRAM represents one example [53–57]. In the medium term, a concerted effort towards developing an open quantum data structure library may be useful for improving our understanding of the types of quantum approaches that may admit practical advantages over the long term.

## 6. Summary

The landscape of quantum advantages considers the benefits of quantum computing technologies relative to existing classical alternatives. An advantage is identified by evidence, which varies according to its theoretical, experimental or operational context. We divide quantum advantages into four classes on the basis of the strength of the quantum advantage (i.e. a polynomial or superpolynomial reduction in complexity) and the complexity of the analogous classical algorithm (i.e. polynomial or superpolynomial). Each class implies differing prospects for achieving experimental and operational advantages in the near term.

Among the four classes of quantum advantage, superpolynomial advantages on classically hard problems appear to present the most viable path to operational advantages. In the biomedical sciences, relevant domain problems in this class include ones related to the quantum simulation of biologically relevant molecules—such as small molecules, protein domains, and nucleic acids—and their chemical quantities. This suggests that the fields of drug development, biochemistry and structural biology may stand to benefit over the near term from targeted proof of principles leveraging hybrid quantum–classical approaches, such as variational quantum simulation.

Quantum advantages may result from a variety of quantum algorithm paradigms over the near term. These include variational quantum simulation, variational quantum machine learning, quantum approximate optimization algorithms and quantum annealing. Already, variational quantum algorithms have shown particularly promising results. This is partly due to their substantial flexibility, which allows them to tackle a wide variety of problems across quantum simulation, quantum machine learning and optimization. Notably, while VQAs may yield superpolynomial advantages on classically hard problems, whether these near-term algorithms can fully capitalize on the computational power afforded by quantum information remains a matter of theoretical investigation (e.g. [424]). Nonetheless, quantum supremacy experiments [10,12] leveraging parametrized quantum circuits—the core quantum component of near-term hybrid quantum–classical algorithms—have already demonstrated the viability of quantum advantages on NISQ devices.

More speculatively, quantum machine learning algorithms yielding advantages in sample complexity (including smaller, polynomial ones) may translate into meaningful empirical advantages in the near to medium term. In particular, polynomial advantages in data resources may allow for the training of quantum machine learning models that exhibit generalization error rates similar to classical machine learning models while also requiring less training data than their classical counterparts. Given the pervasive challenges around generating and processing biological and clinical samples, the experimental validation of sample size advantages may provide a basis for significant operational advantages that may be resilient to improvements in classical hardware capabilities. However, the existence of such advantages in practical settings requires further validation.

Finally, we note that it remains possible that the greatest fruit of research into quantum approaches will be novel quantum inspired classical algorithms. For example, the previously noted framework [183] for the dequantization of QML algorithms [177,178] based on the QLSA has led to the development of classical algorithms that may in the future improve upon existing practical implementations. Similarly, the development of one classical optimization algorithm for constraint satisfaction [425] was inspired by the original QAOA [215] and improved upon its performance. Further, recent work on the value of data in classical computation has extended our understanding around the types of hard problems that may be tractable with high-quality data and classical machine learning techniques [201]. On the hardware side, stiff competition between quantum approaches [10] and their classical counterparts [426–430], and the potential that decoherence may not be tamed, together leave open the possibility that large quantum advantages or fault tolerant quantum computers may not be possible. However, a diverse and growing body of evidence—from recent work on novel, more efficient error correcting codes [114,116], early demonstrations of logical qubits [431–433] and the realization of dynamical topological phases [434,435], to the identification of significant operational quantum advantages in the energy required to perform computations [427,436]—gives much reason for optimism.

## Data Availability

This article has no additional data. Additional information is provided in electronic supplementary material [468].

## Footnotes

^1^
In the context of this work, quantum supremacy [437] is considered a special case quantum advantage.

^2^
While each experiment represents a state-of-the-art demonstration of the capabilities of quantum devices, they are not without limitations. For example, whether the classical simulation performed in [10] constitutes the practical limit of classical computation has been contested [426,438], although recent work has shown substantial advantages in energy consumption for the specific experiment [427,436]. Further, while the superconducting devices in [10,12,13] are programmable, the photonic device in [11] was (i) hard-coded to perform the specific task and (ii) a Gaussian boson sampler (GBS) implementing a limited, non-universal model of quantum computation.

^3^
Given the early stage of quantum hardware and algorithm development, we would like to emphasize that while quantum hardware is now widely accessible, it may nonetheless take many years for *quantum applications* to yield practical benefits in the biomedical sciences. However, the potential of practical advantages nonetheless provides ample motivation for the pursuit of this inherently interdisciplinary research.

^4^
For detailed coverage of topics within the field of QIS, see the following reviews on quantum information theory [439], quantum algorithms [440,441] and quantum hardware [442]. For an exhaustive discussion of near-term quantum algorithms and their prospects, we direct the reader to the recently published paper by Bharti *et al.* [443], alongside the more focused reviews on quantum machine learning [444] and variational quantum algorithms [134]. See the recent review by Eisert *et al.* for a discussion of hardware and software certification and benchmarking [445] and one by Gheorghiu *et al.* for a discussion of the more stringent verification of a quantum computation [446]. While the standard textbook for the QIS field is Nielsen & Chuang [37], for a recent, application-oriented review of quantum algorithms and their circuit implementations, we recommend this overview [447]. For background on some of the topics in biology and medicine we discuss, the following resources on computational approaches in drug discovery [22], phylogenetics [376], medical image segmentation [448], *de novo* assembly [359], biological sequence error correction [415] and biomedical deep learning applications [449] may be of value.

^5^
Bounds are typically expressed using *big **O** notation*. We provide a brief explanation of this and similar notations in the glossary provided in the electronic supplementary material.

^6^
Note that it is convention to take *N* to mean *N* = 2^*n*^. This is due to the exponential state space (known as a Hilbert space) generated by an *n*-qubit quantum superposition.

^7^
Here, the tilde provides an indication of this omission from the lower bound Ω(⋅)

^8^
In practice, we are often interested in the best *available* classical algorithm, which may be identified by a mixed criterion that takes into consideration aspects of the quantum algorithm in question and the intended application. Examples may include (i) the most commonly used practical algorithm for a given application, (ii) the most advanced algorithm implemented for that application, or (iii) the algorithm for which the underlying operational assumptions best comport with the ones required for the comparison being made.

^9^
Recent work suggests that, given certain reasonable assumptions, graph property testing algorithms will only be able to yield polynomial quantum advantages [63,450,451].

^10^
Note that NP-hard refers to a well known *complexity class*, which describes a set of related computational problems. For a definition of the term, see the glossary.

^11^
Indeed, quantum simulation is viewed by many as the ‘killer app’ for quantum computing in a wide variety of fields, including chemistry, material science, and structural biology.

^12^
While a theoretical algorithm may indicate that a quantum advantage exists, it need not be implementable or relevant to a known practical problem.

^13^
See [452] for a review and general framework for benchmarking quantum computers.

^14^
For example, in healthcare, if a quantum advantage were developed and operationalized as part of a software-based medical device (e.g. a medical imaging device incorporating software leveraging a quantum machine learning model), it would not be sufficient for the quantum application to deliver an advantage in the computational sense. Rather, for FDA approval in the United States, the device would be required to demonstrate both safety and efficacy for a specific indication, as defined by a set of pre-determined clinical measures.

^15^
Notably, recent work has shown that error mitigation must be implemented with care as it can decrease the effectiveness of circuit parameter learning in some cases [97].

^16^
The required fault-tolerance threshold depends on a variety of factors, including the hardware error characteristics, connectivity map, and error correction code being used.

^17^
A recent case study [115] found that high constant time overheads represent a significant challenge to implementing Shor’s algorithm on a benchmark cryptography application. As modelled, this overhead is due to magic state distillation [453,454], a process required for error correcting non-Clifford gates (crucial operations required for universal quantum computation). While the overhead of magic state distillation has long been viewed as necessary to FTQC, it is notable that this has recently been called into question (e.g. [455]).

^18^
These numerical simulations of quantum circuits included ones for the quantum Fourier transform, Jordan–Wigner transform, and Grover diffusion operator, each compiled using three connectivity maps representative of existing device architectures: all-to-all with *n*(*n* − 1)/2 edges, a two-dimensional square grid with $\sqrt{n}\times \sqrt{n}$ dimensionality and $2(n-\sqrt{n})$ edges, a ladder with *n*/2 × 2 dimensionality and 3*n*/2 − 2 edges, and a linear nearest neighbour graph with degree-1 terminal qubits and *n* − 1 edges.

^19^
Quantum algorithms yielding this class of advantage often leverage amplitude encoding, or other similarly efficient encodings, requiring of the order of log_2_ *N* qubits.

^20^
In particular, the QLSA embeds the data matrix into the amplitudes of the quantum state, which implies that a quantum state with amplitudes proportional to the values in the data matrix must be prepared in *O*(polylog(*n*)) time [72]. To do this, the QLSA assumes access to a quantum random access memory (QRAM) [53,54,57], an advanced quantum hardware device expected to have similar implementation complexity to FTQC hardware. Additionally, given the efficiency of the amplitude encoding used for the data input, the size of the measured output is limited proportionally (i.e. log_2_ *N*) due to Holevo’s bound [135]. To sample the full solution vector, a polynomial number of samples (and an i.i.d.—independent and identically distributed—assumption over output of the quantum circuit) is required, which would abrogate the superpolynomial speedup.

^21^
It is notable that recent work has indicated that this particular input constraint on the QLSA may be less severe in practice, given a modest assumption [456].

^22^
It is notable that, in addition to the many gradient-based optimizers seen in classical deep learning libraries [457], VQAs may leverage quantum-specific optimizers. In particular, the parameter-shift rule [155,156] represents a favoured gradient-based approach developed expressly for VQAs. Recent work on this optimizer has led to a broadening of the gate sets to which it can be applied [157,158] and the use of adaptive shots [159,160], which leverage information from prior circuit evaluations to reduce the number of measurements required. Other optimizers include a quantum analogue of natural gradient descent [161,162], derivative-free methods [163] and Bayesian approaches [164].

^23^
Classical numerical simulations of VQEs and related approaches have been performed for larger systems; for one example, see [458].

^24^
In particular, assuming the matrix is low-rank [177,183], the classical algorithm could be used to replace a core subroutine of the quantum recommendation system algorithm [52], reducing the advantage from superpolynomial to polynomial.

^25^
While the unification of quantum metrology and QML appears unexplored for biological and clinical research, recent work on quantum algorithms for medical imaging represents a movement in this direction [459].

^26^
It is interesting that classical neural networks were conceived as inherently nonlinear while the linearity of quantum mechanics is well established (indeed, it was shown in 1998 that were quantum mechanics to be nonlinear, quantum computers could solve NP-complete problems in general) [460]. This apparent incompatibility does not represent a significant challenge, however. Multiple approaches to QNN design allow for nonlinearity to be approximated to yield the required transformation. These include continuous-variable QNNs [193] and ones leveraging ancilla qubits to implement reversible gates that emulate nonlinearity [461,462].

^27^
It is notable that changing the data encoding strategy (e.g. from basis encoding to amplitude encoding) changes the resulting quantum kernel [202].

^28^
This makes the somewhat strong assumption that *p* grows only logarithmically with respect to the problem instance. In practice, it seems likely that polynomial or even exponential scaling may be required to solve certain problems.

^29^
The QAOA algorithm also motivated the development of a novel classical algorithm [425], which improved upon the quantum lower bound for approximation of constraint satisfaction problems.

^30^
Like the programmable devices used in the recent quantum supremacy experiments [10,12,13], commercial quantum annealers also use a superconducting qubit architecture, with some differences in qubit implementation.

^31^
The computational model implemented by practical quantum annealers is believed to be non-universal, unlike the adiabatic model [463,464]. This limitation is due to the finite temperature of the hardware during computation; in principle, a quantum annealer operating at zero kelvin could be universal.

^32^
Quantum tunnelling is a uniquely quantum mechanical effect that was previously thought to be a potential source of advantage for QA devices.

^33^
Each method has different advantages with respect to molecular specificity and spatiotemporal resolution. The appropriate form of spectroscopy varies by application.

^34^
Simulation of electron dynamics is distinct from MD, which is concerned primarily with the motion of the atomic coordinates.

^35^
We also note that a distinct algorithm called quantum imaginary time evolution (QITE) was recently proposed, which may also be promising for near-term hardware [465].

^36^
A trade-off between read length and base error rates with short and long read sequencing technologies yields complementary characteristics. This makes hybrid approaches to *de novo* assembly advantageous (this can be viewed as a close sibling of the well-documented trade-off between depth and coverage in short read sequencing data [466]). Long read technologies allow for the scaffolding of homopolymeric and repetitive regions that are beyond the length of short read sequences; short read technologies generate many overlapping reads of the sequenced regions providing a large sample size (the depth of coverage for modern short read sequencers ranges from 3 × 10^1^ to of the order of 10^4^) and greater statistical confidence for each base call (each of which can be treated as an individual hypothesis test).

^37^
It is notable that phylogenetic tree inference has taken centre stage in the COVID-19 pandemic with tools such as Nextstrain [467], which has been used to monitor the evolution of SARS-nCoV-2.

^38^
It is necessary to assume that the unseen dataset is i.i.d. to the data used to train the machine learning model.

^39^
While this may not be true in the NISQ era, it may well be the case for FTQC devices and is certainly the case for classical HPCs.

^40^
It is notable that the existence of polynomial advantages in sample complexity is an active research area in the QIS field.
